# A Bioinformatics-Driven ceRNA Network in Stomach Adenocarcinoma: Identification of Novel Prognostic mRNA-miRNA-lncRNA Interactions

**DOI:** 10.3390/medsci13040214

**Published:** 2025-10-01

**Authors:** Ebtihal Kamal, Zainab Mohammed Mahmoud Omar, Ayman Geddawy, Ahmad A. A. Omer

**Affiliations:** 1 Department of Basic Medical Sciences, College of Medicine, Prince Sattam Bin Abdulaziz University, Alkharj 16278, Saudi Arabiaggeddawy@gmail.com (A.G.); 2 Department of Pharmacology, Faculty of Medicine, Al-Azhar University, Assiut 71511, Egypt; 3 Department of Pharmacology, Faculty of Medicine, Minia University, Minia 61511, Egypt; 4 Department of Surgery, Faculty of Medicine, Prince Sattam Bin Abdul Aziz University, Alkharj 16278, Saudi Arabia; ahmedsilik@gmail.com

**Keywords:** stomach adenocarcinoma, prognostic biomarker, ceRNA network, bioinformatics

## Abstract

Background: Stomach adenocarcinoma is a major contributor to worldwide mortality and significantly impacts life expectancy. The main objective of the current study was to identify a prognostic biomarker for stomach adenocarcinoma to advance translational medicine and improve patient outcomes. Method: various databases (GEPIA, UALCAN, miRNet, StarBase, and Kaplan Meier plotter) bioinformatics tools (cytoscape) and were used in this study. Results: Ten novel unfavorable prognosis-associated *genes* were identified. In addition, 41 potential miRNAs were predicted. ELAVL3-hsa-mir-29a-3p and CALCR-hsa-mir-29a-3p were identified as the two critical networks in the oncogenesis of stomach adenocarcinoma via bioinformatics analysis. Subsequently, the binding of lncRNAs to hsa-mir-29a-3p was predicted utilizing the starBase and miRNet databases. Following the execution of both expression and survival analyses for the predicted lncRNAs, it was determined that only one lncRNA, KCNQ1OT1, exhibited significant overexpression in stomach adenocarcinoma, and its elevated expression was associated with an unfavorable prognosis. Subsequently, we constructed a triple ceRNA network involving mRNA, miRNA, and lncRNA, which is associated with the prognosis of stomach adenocarcinoma. Conclusions: In summary, the current study provides an extensive ceRNA network that highlights novel prognostic biomarkers for stomach adenocarcinoma.

## 1. Introduction

Gastric cancer (GC), with stomach adenocarcinoma (STAD) being a prominent subtype, continues to pose a significant global health challenge despite progress in treatment methodologies. It is associated with a high mortality rate and unfavorable prognosis, making it the fifth most frequently diagnosed cancer and a leading cause of cancer-related deaths worldwide, as reported by GLOBOCAN 2020 [1]. The risk of STAD is increased by chronic gastropathies and Epstein-Barr virus (EBV) infections, as well as by demographic and lifestyle factors [2,3]. Moreover, genetic predisposition plays a significant role in the development and progression of STAD, with increasing scientific attention directed towards long non-coding RNAs (lncRNAs).

STAD treatment modalities include surgery, radiotherapy, and anticancer drugs that may be used as neoadjuvant, adjuvant, or palliative therapies [4,5]. However, current medications have little efficacy in treating advanced STAD. Therefore, new therapeutic strategies are urgently needed [6]. Immunotherapy is more effective than standard treatment, resulting in longer survival times and improved outcomes [7,8]. The advanced pharmacotherapy of STAD presents a rapidly evolving landscape, with the addition of targeted/immune therapies, including anti-HER2 and FGFR2 inhibitors [9]. Despite advances in carcinogenesis research and new therapeutic strategies, patients with STAD have a poor prognosis, and precision medicine for STAD remains challenging [10,11]. The identification of additional molecular markers associated with STAD will aid in managing patients and improving their prognosis. Elucidating the complex mechanisms underlying STAD pathogenesis and identifying potential diagnostic and prognostic biomarkers may facilitate the development of effective therapeutic strategies and enhance patient outcomes. Salmena et al. proposed the ceRNA hypothesis, which posits that ncRNAs, such as lncRNAs, can modulate gene expression by competitively binding to shared miRNAs with mRNAs [12]. Competing endogenous RNAs (ceRNAs), encompassing both long non-coding RNAs (lncRNAs) and circular RNAs (circRNAs), can modulate expression of target *genes* by competing with mRNAs for identical microRNA response elements (MREs) [12,13].

Recent studies have extensively investigated the roles of ceRNA regulatory networks in various human malignancies. Researchers have used ceRNA networks to identify prognostic markers in glioblastoma [14,15,16], thyroid cancer [17], pancreatic cancer [18], hepatocellular carcinoma [15], and breast cancer [19].

We aimed to build an extensive “mRNA-miRNA-lncRNA” ceRNA network to identify new prognostic biomarkers for STAD that contribute to precision medicine by utilizing cutting-edge bioinformatics technologies to identify important molecular connections associated with patient outcomes.

Although the ceRNA regulatory network has been studied in STAD in prior research [20,21], this study adopts a different methodology through analysis and integration of results using different databases (GEPIA, UALCAN, Kaplan-Meier plotter, miRNet, and StarBase). It incorporates modern analytics to construct a unique “mRNA-miRNA-lncRNA” framework that allows for the discovery of ceRNA components with prognostic value in STAD, linking molecular interactions to patient outcomes and enhancing precision medicine.

## 2. Materials and Methods

**Figure 1:**
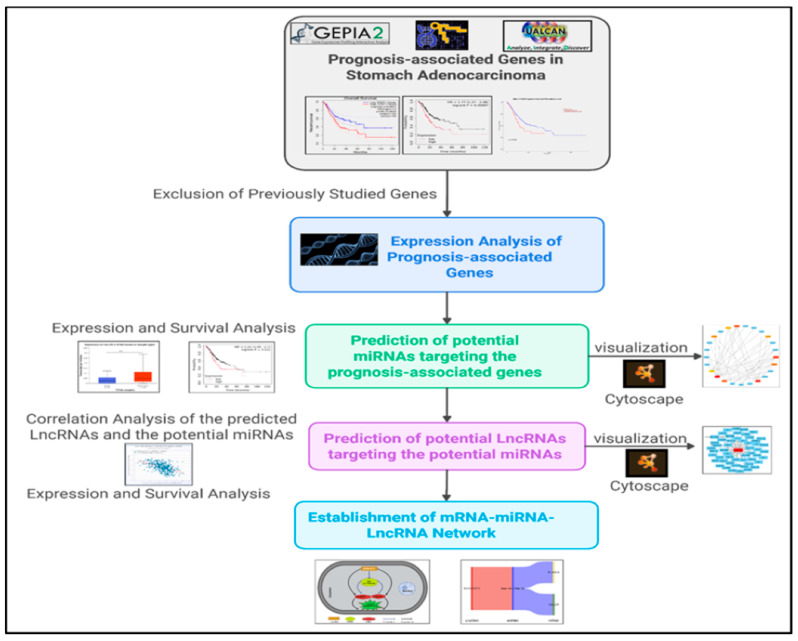
Workflow of the study.

The steps of the study were summarized in the workflow presented in Figure 1.

### 2.1. GEPIA Database

GEPIA is a newly developed interactive online platform designed for the analysis of RNA sequencing expression information derived from the Cancer Genome Atlas (TCGA) and Genotype-Tissue Expression (GTEx) projects [22]. GEPIA was used to identify the *genes* most correlated with overall survival (OS) and disease-free survival (DFS) in patients with STAD. GEPIA is available at http://gepia.cancer-pku.cn/detail.php (accessed on 12 June 2024).

### 2.2. UALCAN Database

The TCGA is an extensive, accessible, and interactive online platform for analyzing cancer data [23]. We used the expression module of UALCAN to study the expression profiles of the prognosis-associated *genes* in TCGA STAD and normal tissues. UALCAN is available at http://ualcan.path.uab.edu (accessed on 18 June 2024).

### 2.3. MiRNet Database

MiRNet (http://www.mirnet.ca/, accessed on 16 July 2024), a user-friendly online platform for miRNA-related research, was utilized to predict the potential miRNA binding regions on mRNAs and to anticipate potential lncRNA interactions with miRNAs [24].

### 2.4. StarBase Database

StarBase is an open-source database specifically designed for the analysis of non-coding RNA interactions, utilizing data derived from CLIP-seq, degradome-seq, and RNA-RNA interactome datasets. It was developed to facilitate expression correlation analysis for mRNA-miRNA and miRNA-lncRNA pairs in multiple cancer types [25]. R < −0.1 indicates negative correlation, and a *p* value < 0.05 was established as the threshold for significant correlation [25]. The miRNA expression levels in the STAD were also assessed using StarBase. StarBase is available at http://starbase.sysu.edu.cn/ (accessed on 18 July 2024).

### 2.5. Kaplan-Meier Plotter

The Kaplan-Meier plotter database can be used to evaluate the impact of miRNAs and *genes* on survival across 21 cancer types [26]. The predictive significance of potential miRNAs in STAD was assessed using the Kaplan-Meier plotter (http://kmplot.com/analysis/, accessed on 16 July 2024). Each miRNA of interest was initially submitted to the database. Based on the median expression value, all cases were categorized into low- and high-expression groups.

### 2.6. Visualization Tools

Cytoscape (version 3.10.3) is a powerful, open-source software platform used to visualize complex networks [27]. It is widely used in bioinformatics to visualize molecular interaction networks and biological pathways. Sub-analyses were performed to establish mRNA-miRNA and miRNA-lncRNA regulatory networks, and Cytoscape was used to visualize the relationships between miRNAs and their corresponding mRNAs and lncRNAs.

Bioinformatics.cn.com is a freely accessible, easy-to-use web server, available at https://www.bioinformatics.com.cn/en (accessed on 5 August 2024). It integrates more than 120 commonly used data visualization and graphing functions, including heatmaps, Venn diagrams, volcano plots, bubble plots, and scatter plots. We used the Sankey diagram from Bioinformatics.cn.com to visualize the interactions between mRNA, miRNAs, and lncRNAs.

### 2.7. Statistical Examination

Most statistical analyses were conducted using online bioinformatics tools. *p*-values and log-rank tests were obtained using GEPIA expression analysis. *p*-values from the Kaplan-Meier plotter survival analysis were adjusted for the false discovery rate. Statistical significance was set at *p* < 0.05.

In mirNet, the network-based visualization, particularly for miRNA-target interactions, a hypergeometric test is employed to determine if the observed number of interactions between miRNAs and their target *genes* is greater than expected by chance.

The correlation analysis performed in the StarBase database primarily involves studying the correlation between lncRNAs, miRNAs, and mRNAs. This analysis typically includes various statistical methods, such as Pearson correlation analysis, to assess the strength and direction of the correlation between these RNA types.

## 3. Results

### 3.1. Identification of 10 Novel Prognosis-Associated Genes in Stomach Adenocarcinoma

The *genes* linked to both OS and DFS in patients with STAD were identified using GEPIA. Tables S1 and S2 list the top 500 OS-associated *genes* and the 500 DFS-associated *genes*, respectively, based on *p* < 0.05. Through the intersection of *genes* linked to OS and DFS, we identified 90 OS- and DFS-associated *genes*. Upon reviewing the available literature and prior studies, we identified ten *genes* (CALCR, CFHR1, CPT1C, ELAVL3, FLJ16779, MYOZ3, NALCN, TIGD6, TPST1, and ZNF474) that have not yet been examined or have had limited studies for their predictive significance in STAD. The prognostic values (OS and DFS) of the 10 *genes* using GEPIA (Figure S1). The results suggested that high expression of the ten *genes* indicated poor prognosis in patients with STAD.

The Kaplan-Meier plotter was used to compare OS between the two groups (upregulated and downregulated expression). We found that all the previously identified prognosis-associated *genes* by GEPIA were also identified as prognosis-associated *genes* in the Kaplan-Meier plotter (Figure S2). Therefore, these ten *genes* were considered as potential novel prognostic biomarkers for STAD, and further studies were concentrated on these *genes*.

### 3.2. Identification of the Upregulated Prognosis-Associated Genes

Subsequently, we assessed the expression levels of the ten novel *genes* linked to STAD. We identified the difference in their expression profiles in TCGA-STAD and normal tissues via the UALCAN database, revealing that all *genes* were highly expressed in STAD samples compared to normal samples (Figure S3).

### 3.3. Prediction of Potential miRNAs Binding to Novel Prognosis-Associated Genes

**Figure 2:**
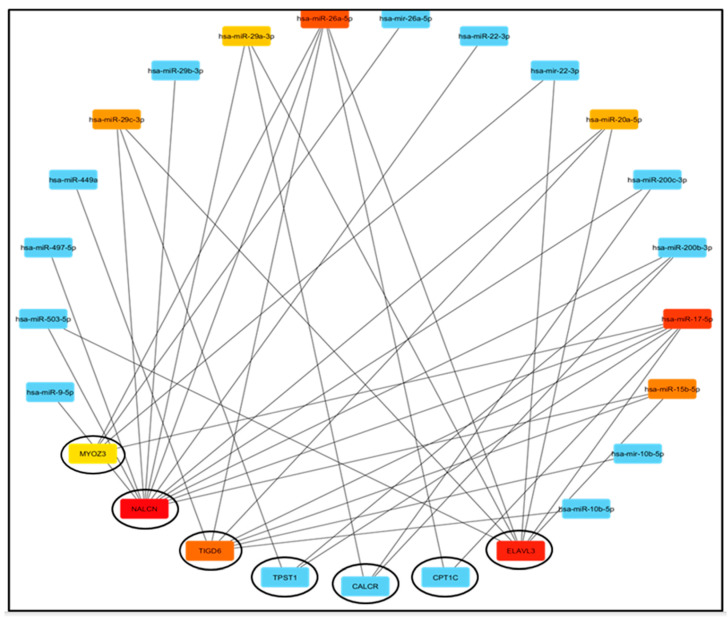
Construction of NALCN/ELAVL3/TPST1/CALCR/MYOZ3/TIGD6 and CPT1C-miRNA network by miRNet database and visualization by Cytoscape software (v3.10.3).

Next, we predicted the upstream regulatory miRNAs of the ten novel prognosis-associated *genes* using miRNet, a comprehensive miRNA study-associated database. Only seven of the ten prognosis-associated *genes* had significantly associated miRNAs. A total of forty-one mRNA-miRNA pairs were identified, and an interactive network was developed using Cytoscape software for better visualization and detailed mRNA-miRNA pairs (Figure 2).

The classical mechanism of miRNA action in negatively regulating gene expression suggests an inverse relationship between expected mRNA-miRNA interactions. Consequently, we used the StarBase database to study the correlation between prognosis-associated *genes* and miRNAs in STAD. Among the forty-one interactions, twenty mRNA-miRNA pairs were predicted as significant (Figure S4).

**Figure 3:**
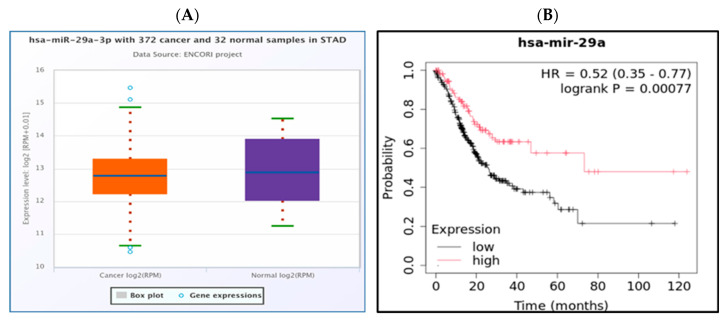
Expression of has-mir-29a-3p in STAD compared to normal tissue. The bioinformatics analysis presented in this figure was performed using starBase (http://starbase.sysu.edu.cn) (**A**). Prognostic value of the downregulated has-mir-29a-3p expression in STAD. The bioinformatics analysis presented in this figure was performed using Kaplan Meier plotter (http://kmplot.com/analysis/) (**B**).

Hypothetically, miRNAs that bind to oncogenic *genes* should be downregulated in STAD. Expression analysis of significant miRNAs was performed using the StarBase database, revealing that only one significant miRNA, hsa-miR-29a-3p, exhibited downregulated expression in STAD (Figure 3A). The prognostic significance of this potential miRNA in STAD was assessed using the Kaplan-Meier plotter database. We verified that hsa-mir-29a-3p was downregulated, and its downregulation is correlated with an unfavorable prognosis (Figure 3B). Through the integration of both expression and survival analyses, we found that hsa-mir-29a-3p emerged as a potential miRNA in STAD.

### 3.4. Prediction of Key LncRNA Binding to Potential miRNA

**Figure 4:**
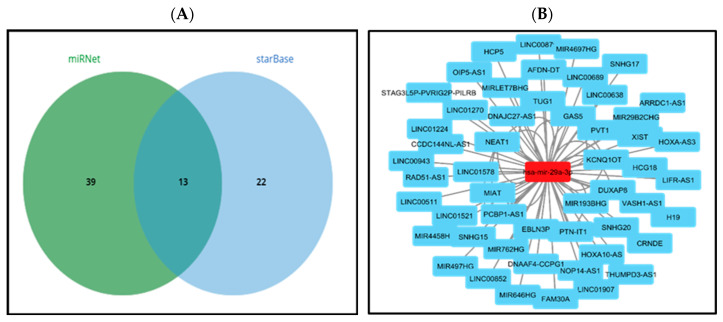
Prediction of upstream lncRNAs potentially binding to hsa-mir-29a-3p (**A**). miRNA–lncRNA regulatory network by miRNet using Cytoscape software (v3.10.3) (**B**).

Previous studies have demonstrated that lncRNAs can interact with miRNAs, thereby facilitating the regulation of target gene expression and executing biological functions. Therefore, the miRNet and starBase databases were utilized to forecast potential lncRNAs that could interact with hsa-mir-29a-3p. Fifty-two and thirty-five lncRNAs were identified as potential targets of hsa-mir-29a-3p by miRNet and starBase, respectively. Thirteen lncRNAs (MIR29B2CHG, MIR4458HG, STAG3L5P-PVRIG2P-PILRB, VASH1-AS1, DNAAF4-CCPG1, MIRLET7BHG, XIST, GAS5, THUMPD3-AS1, EBLN3P, KCNQ1OT1, NEAT1, and OIP5-AS1) bind to hsa-mir-29a-3p, were consistently identified in both the miRNet and starBase databases (Figure 4A). These lncRNAs were selected for further investigation. The regulatory network between miRNA and lncRNA was developed utilizing Cytoscape software to enhance visualization (Figure 4B).

Considering the ceRNA hypothesis, LncRNAs targeting hsa-mir-29a-3p are likely to be oncogenic in STAD. We found that both KCNQ1OT1 and OIP5-AS1 LncRNAs had a significant negative correlation with hsa-mir-29a-3p (Table 1).

**Figure 5:**
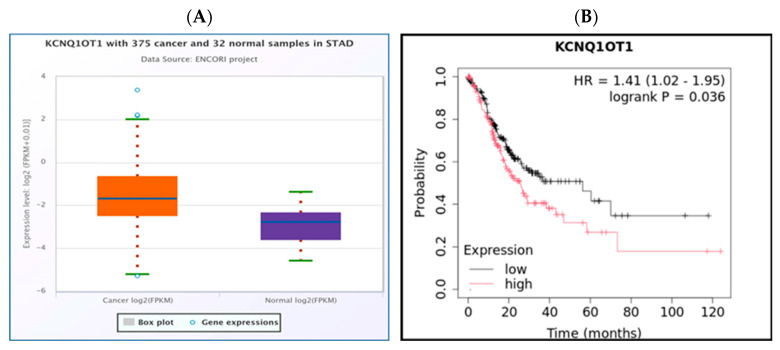
Expression of KCNQ1OT1 lncRNA in STAD. The bioinformatics analysis presented in this figure was performed using starBase (http://starbase.sysu.edu.cn) (**A**). Survival analysis of KCNQ1OT1 expression in STAD. The bioinformatics analysis presented in this figure was performed using Kaplan Meier plotter (http://kmplot.com/analysis/) (**B**).

**Figure 6:**
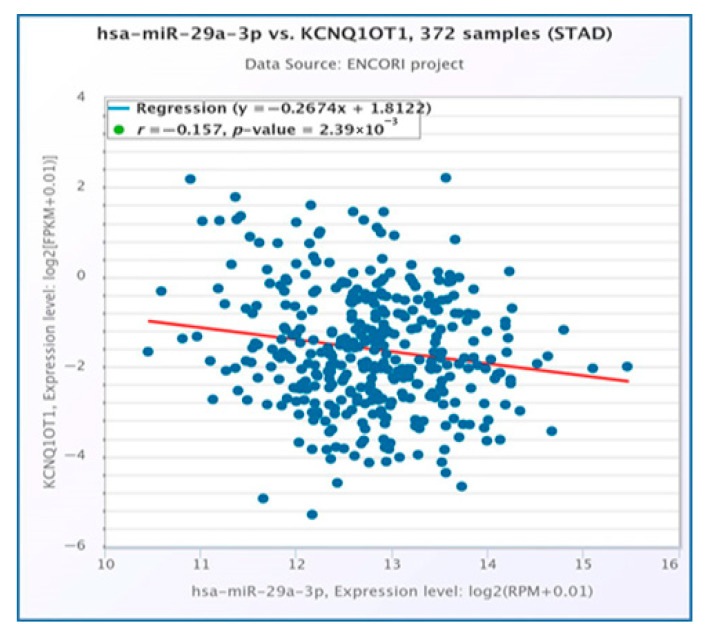
Correlation between hsa-mir-29a-3p and KCNQ1OT1. The bioinformatics analysis presented in this figure was performed using starBase (http://starbase.sysu.edu.cn).

Through the integration of expression and survival analyses, we identified a single lncRNA, KCNQ1OT1, which was significantly upregulated in STAD using the StarBase database (Figure 5A). Furthermore, its upregulation was associated with poor prognosis as determined by the Kaplan-Meier plotter (Figure 5B). Furthermore, we found that KCNQ1OT1 was significantly negatively correlated with hsa-mir-29a-3p (Figure 6). The current findings support that KCNQ1OT1 is upregulated in STAD, correlated with poor prognosis significantly negatively correlated with hsa-mir-29a-3p. This might be a potential lncRNA that binds to the previously identified miRNA, hsa-mir-29a-3p.

### 3.5. Identification of the Upstream miRNA-lncRNA Network of the Predicted Novel mRNAs in Stomach Adenocarcinoma

Utilizing the ceRNA mechanism, we developed a novel mRNA-miRNA-lncRNA network in STAD. Initially, we identified the upstream miRNAs of NALCN, CPT1C, ELAVL3, MYOZ3, TPST1, TIGD6, and CALCR through miRNet. This analysis resulted in the prediction of forty-one miRNA-mRNA pairs (Figure 2). Moreover, we performed an expression correlation analysis for the miRNA-mRNA pairs in STAD and found that only twenty pairs presented significantly negative relationships in their expression (Figure S4). Expression and survival analyses predicted that only one miRNA (hsa-mir-29a-3p) was significantly downregulated in STAD and correlated with an unfavorable prognosis (Figure 3). Subsequently, we utilized the miRNet and starBase databases to predict the upstream lncRNAs associated with the potential miRNA (hsa-mir-29a-3p). Our analysis identified thirteen intersecting lncRNAs as potential targets of hsa-mir-29a-3p; only KCNQ1OT1 and OIP5-AS1 were significantly negatively correlated with hsa-mir-29a-3p (Table 1).

**Figure 7:**
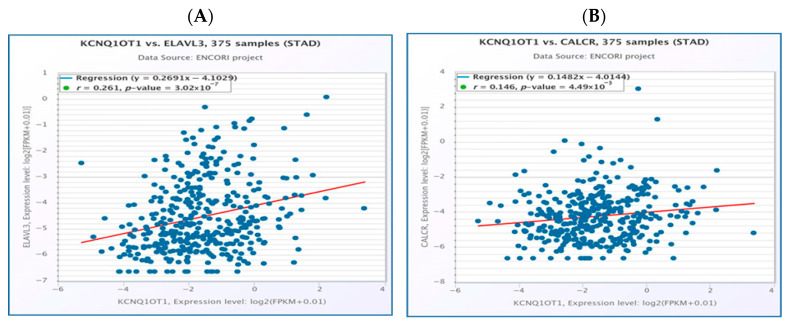
Correlation between KCNQ1OT1 and ELAVL3 in stomach adenocarcinoma (**A**). KCNQ1OT1has a significant positive correlation with CALCR expression in stomach adenocarcinoma (**B**). The bioinformatics analysis presented in this figure was performed using starBase (http://starbase.sysu.edu.cn).

One potential lncRNA, KCNQ1OT1, and one potential miRNA (hsa-mir-29a-3p) formed an miRNA–lncRNA. The ceRNA hypothesis posits a positive correlation between the mRNA and lncRNA expression. The StarBase database was used to investigate the expression correlation of the three mRNA-LncRNA pairs (KCNQ1OT1-NALCN, KCNQ1OT1-ELAVL3, and KCNQ1OT1-CALCR). Significant positive expression correlations of these mRNA-LncRNA pairs were detected for both ELAVL3 and CALCR (Figure 7A,B).

**Figure 8:**
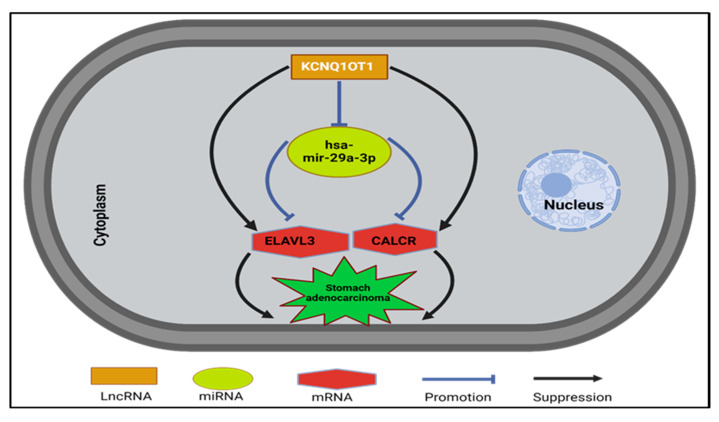
The established mRNA-miRNA-LncRNA competing endogenous RNA (ceRNA) triple network is associated with the progression and prognosis of STAD. Created in BioRender. Kamal, E. (2025) https://BioRender.com/f15v724.

**Figure 9:**
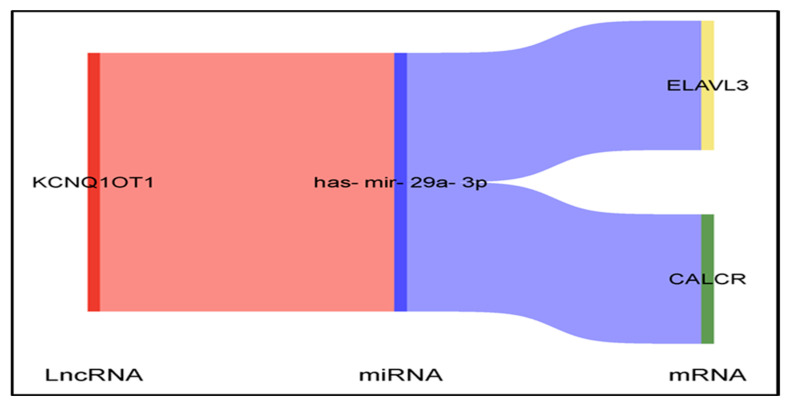
Sankey diagram showing novel lncRNA–miRNA–mRNA interactions in STAD.

Through the integration of our findings from this in silico study, We have identified a significant mRNA-miRNA-lncRNA triple regulatory network associated with the prognosis of STAD (Figure 8 and Figure 9).

## 4. Discussion

In this study, we identified 500 *genes* associated with OS and 500 *genes* associated with DFS in patients with STAD (Tables S1 and S2). Subsequently, we identified 90 *genes* that were common to both OS and DFS associations. A comprehensive literature review was conducted using the PubMed database to ensure the exclusion of *genes* previously studied in relation to STAD. Our search employed specific terms such as “gene names,” “stomach adenocarcinoma,” and “predictive significance,” utilizing Boolean operators (AND/OR) to refine the search results. Inclusion criteria focused on peer-reviewed studies that examined the role of specific *genes* in STAD, particularly those discussing predictive significance and original research findings, while exclusion criteria eliminated articles that were not directly related to STAD, those that had already established the predictive significance of the *genes* in question, and reviews lacking original data. This systematic approach allowed for the identification of a list of previously studied *genes*, ensuring that the final selection of ten novel *genes* from an initial pool of ninety was based on a thorough understanding of the existing literature, thereby highlighting *genes* that had not yet been examined or had limited studies on their predictive significance in STAD.

Ten *genes*, namely CALCR, CFHR1, CPT1C, ELAVL3, FLJ16779, MYOZ3, NALCN, TIGD6, TPST1, and ZNF474, have been identified as novel or minimally studied in relation to STAD, and were identified as prognosis-associated *genes* in STAD. The CALCR gene encodes the calcitonin receptor, which is recognized for its significant roles in cancer biology, particularly in the regulation of cell proliferation, apoptosis, and migration [28,29], while there is a scarcity of research specifically associating CALCR with STAD in the current literature. CFHR1 (Complement Factor H-Related Protein 1) is a protein that plays a role in the immune system, particularly in the regulation of the complement pathway, which is crucial in immune responses [30]. Some studies have indicated that CFHR1 may be expressed in various cancers, including LUAD [31] and bile duct carcinoma [32], but its role in STAD is unclear.

Carnitine palmitoyltransferase 1C (CPT1C) has become an important element in cancer research, especially concerning tumor survival and progression [33]. CPT1C plays a role in the regulation of fatty acid oxidation (FAO), a process essential for energy production in cancer cells [34]. One study identified that elevated expression of CPT1C, induced by hypoxic conditions, was significantly correlated with poor prognosis and facilitated the proliferation of GC cells [35]. ELAVL3 (ELAV-like RNA-binding protein 3) is increasingly recognized for its involvement in cancer, particularly neuroendocrine tumors [36]. However, direct investigations of ELAVL3 in STAD are limited.

A recent study has identified a predictive signature of FLJ16779 in STAD, facilitating the stratification of patients into high- and low-risk groups. This stratification is essential for customizing treatment strategies and enhancing patient outcomes. Although the precise mechanisms by which FLJ16779 influences STAD remain under investigation [37]. NALCN (Sodium Leak Channel Non-Selective) has been identified as a tumor suppressor gene [38,39]. The expression and functional role of NALCN in STAD have not been comprehensively examined in the current scientific literature. TIGD6 (Tigger Transposable Element Derived 6), there is no study linking this gene to STAD Tyrosylprotein Sulfotransferase 1 (TPST1) plays a crucial role in the post-translational modification of proteins through the process of tyrosine sulfation [40].

Studies suggest that TPST1 may function as a prognostic biomarker in various cancers [41,42]. Despite its involvement in tyrosine sulfation and interactions with oncogenic pathways, which render it a potential gene for cancer research, there is currently no study available that associates it with STAD in the existing literature.

ZNF474 (Zinc Finger Protein 474) may have a role in STAD, although its specific contributions to the disease remain inadequately defined.

Our findings demonstrate that elevated expression levels of CALCR, CFHR1, CPT1C, ELAVL3, FLJ16779, MYOZ3, NALCN, TIGD6, TPST1, and ZNF474 are correlated with a poor prognosis in patients with STAD (Figures S1 and S2). Furthermore, all ten *genes* were significantly upregulated in STAD (Figure S3). mRNAs can engage in competitive interactions with lncRNAs by binding to shared miRNAs. This interaction allows for the prediction of potential miRNAs associated with *genes* such as NALCN, CPT1C, ELAVL3, MYOZ3, TPST1, TIGD6, and CALCR, as well as the lncRNAs linked to these miRNAs. An initial identification of forty-one miRNAs associated with NALCN, CPT1C, ELAVL3, MYOZ3, TPST1, TIGD6, and CALCR was performed utilizing the miRNet database (Figure 2). Given the interaction of miRNAs with mRNA and the hypothesized oncogenic functions of the *genes* associated with prognosis, it is anticipated that miRNAs with tumor-suppressive potential would exhibit a negative correlation with NALCN, CPT1C, ELAVL3, MYOZ3, TPST1, TIGD6, and CALCR.Through correlation analysis, we identified twenty potential mRNA-miRNA interaction pairs in STAD (Figure S4). Following expression and survival analyses, only one of these twenty pairs was selected for further investigation (Figure 3).

Hsa-miR-29a-3p has been implicated in the regulation of gene expression, particularly those *genes* involved in cellular processes such as proliferation, migration, and apoptosis [43]. In the context of breast cancer and hepatocellular carcinoma, hsa-miR-29a-3p has been recognized as a critical factor in tumor progression [44,45]. Consistent with our findings, a study reported that the expression levels of hsa-miR-29a-3p in GC tissue samples were significantly reduced compared to those in adjacent normal tissue samples [46].

The interaction of lncRNAs with hsa-miR-29a-3p was predicted utilizing the miRNet and starBase databases. A total of thirteen lncRNAs, namely MIR29B2CHG, MIR4458HG, STAG3L5P-PVRIG2P-PILRB, VASH1-AS1, DNAAF4-CCPG1, MIRLET7BHG, XIST, GAS5, THUMPD3-AS1, EBLN3P, KCNQ1OT1, NEAT1, and OIP5-AS1, were consistently identified in association with hsa-miR-29a-3p across both databases (Figure 4A). The selected lncRNAs were subjected to further analysis. A miRNA–lncRNA regulatory network was constructed utilizing Cytoscape software to enhance visualization (Figure 4B). Subsequently, the StarBase database was employed to examine the correlation between the potential miRNA and the predicted thirteen lncRNAs. It was observed that both KCNQ1OT1 and OIP5-AS1 lncRNAs exhibited a significant negative correlation with hsa-mir-29a-3p (Table 1). According to the ceRNA hypothesis, candidate lncRNAs of hsa-mir-29a-3p are expected to act as potential oncogenic lncRNAs in STAD. Among all predicted lncRNAs, only KCNQ1OT1 was found to be significantly upregulated in STAD (Figure 5A), and this upregulation was associated with poor prognosis (Figure 5B). Subsequently, we utilized the StarBase database to examine the expression correlation of the three mRNA-lncRNA pairs (KCNQ1OT1-NALCN, KCNQ1OT1-ELAVL3, and KCNQ1OT1-CALCR). Significant positive correlations were identified for both ELAVL3 and CALCR (Figure 7A,B).

KCNQ1OT1, a long non-coding RNA with oncogenic properties, has been linked to pivotal signaling pathways that drive cancer progression. Among these, the Wnt/β-catenin pathway is particularly significant, as it plays a crucial role in regulating cell proliferation and migration [47,48,49,50]. KCNQ1OT1 significantly contributes to progression of GC via the KCNQ1OT1/miR-378a-3p/RBMS1 pathway, which is considered as a crucial prognostic biomarker and a potential therapeutic target for GC [51,52,53].

Through the integration of mRNA-miRNA and miRNA-lncRNA interactions, we identified a specific network (ELAVL3/CALCR-hsa-mir-29a-3p-KCNQ1OT1) associated with the prognosis of STAD (Figure 8 and Figure 9). This network aligns with the previously established roles of hsa-mir-29a-3p and KCNQ1OT1 in various cancers.

The ELAVL3/CALCR-hsa-mir-29a-3p-KCNQ1OT1 network significantly influences the hallmarks of STAD through a complex interplay of regulatory mechanisms. Central to this network, hsa-mir-29a-3p functions as a tumor suppressor, typically inhibiting cell proliferation and metastasis by targeting key oncogenic mRNAs [39]. However, the upregulation of KCNQ1OT1 in STAD acts as a sponge for hsa-mir-29a-3p, effectively sequestering it and preventing it from exerting its suppressive effects [54].

The dysregulation results in the overexpression of ELAVL3 and CALCR, both of which are linked to increased tumor growth and metastatic potential. Specifically, ELAVL3 stabilizes mRNAs of potent oncogenes, thereby promoting cell survival and proliferation [36], while CALCR activates signaling pathways that facilitate invasion and migration [28,55]. This multifaceted influence emphasizes the potential of targeting this network as a therapeutic strategy to improve outcomes for patients with STAD.

Antisense oligonucleotides (ASOs) are short, synthetic strands of nucleic acids designed to bind to specific RNA sequences, thereby modulating gene expression [56]. ASOs can inhibit the expression of KCNQ1OT1 by binding to its RNA transcript, thereby preventing its interaction with target miRNAs or mRNAs. This interference has the potential to disrupt the ceRNA network, which may lead to the restoration of tumor suppressor gene expression that KCNQ1OT1 might be repressing.

Given the association of KCNQ1OT1 with an unfavorable prognosis in stomach adenocarcinoma, the silencing of KCNQ1OT1 using ASOs may result in reduced tumor growth and improved patient outcomes. The interactions within ceRNA networks are not strictly linear, as other non-coding RNAs can also compete for miRNA binding, thereby complicating the regulatory landscape [57].

Moreover, the activity of miRNAs can vary greatly depending on the cellular context [58] and environmental factors [59]. This variability can influence how effectively a miRNA can regulate its target mRNAs. The expression levels of lncRNAs, miRNAs, and mRNAs can differ across various tissues, affecting the dynamics of ceRNA interactions [60]. This tissue specificity is critical for understanding the biological relevance of these networks in different cancer types and stages.

## 5. Limitations

While these findings offer novel insights, they come with certain limitations. To confirm the functional relevance of the identified ceRNA interactions, experimental validation is necessary. Although functional enrichment analysis was not conducted for practical reasons, it could be considered in future research to emphasize the biological roles of the identified *genes*. Additionally, we recognize the absence of correlation analysis between novel ceRNA biomarkers and the clinical and pathological features of STAD, which may have diagnostic and prognostic implications. Furthermore, more research is needed to clarify the molecular roles of less-characterized *genes* like FLJ16779 and TIGD6 in regulating mRNA stability and gene expression pathways that contribute to tumor growth and metastasis.

## 6. Conclusions

This study established a critical framework for understanding STAD’s ceRNA regulatory network in STAD. Although further experimental confirmation is required, these findings highlight interesting biomarkers for diagnostic and therapeutic applications, providing new insights into STAD pathophysiology.

## Figures and Tables

**Table 1:** Correlation between hsa-mir-29a-3p and lncRNA pairs identified by the StarBase database.

miRNAs	lncRNAs	Correlation	*p*-Value
hsa-mir-29a-3p	MIR29B2CHG	r = 0.076	1.042 × 10^−1^
hsa-mir-29a-3p	MIR4458HG	r = −0.008	8.75 × 10^−1^
hsa-mir-29a-3p	STAG3L5P-PVRIG2P-PILRB	r = −0.010	8.53 × 10^−1^
hsa-mir-29a-3p	VASH1-AS1	r = 0.120	2.05 × 10^−2^
hsa-mir-29a-3p	DNAAF4-CCPG1	r = −0.034	5.12 × 10^−1^
hsa-mir-29a-3p	MIRLET7BHG	r= 0.082	1.15 × 10^−1^
hsa-mir-29a-3p	XIST	r = 0.048	3.58 × 10^−1^
hsa-mir-29a-3p	GAS5	r = 0.048	3.53 × 10^−1^
hsa-mir-29a-3p	THUMPD3-AS1	r = −0.053	3.04 × 10^−1^
hsa-mir-29a-3p	EBLN3P	r = −0.085	1.02 × 10^−1^
hsa-mir-29a-3p	KCNQ1OT1	r = −0.157	2.39 × 10^−3^
hsa-mir-29a-3p	NEAT1	r = 0.045	3.83 × 10^−1^
hsa-mir-29a-3p	OIP5-AS1	r = −0.161	1.89 × 10^−3^

## Data Availability

The original contributions presented in this study are included in the article/Supplementary Material. Further inquiries can be directed to the corresponding author.

## Supplementary Materials

The following are available online at https://www.mdpi.com/article/10.3390/medsci13040214/s1, Figure S1: Expression of the 10 novel genes in stomach adenocarcinoma and their prognostic value, using GEPIA database. Figure S2: Expression of the 10 novel genes in stomach adenocarcinoma and their prognostic value using the Kaplan Meier Plotter database., Figure S3: The mRNA expression levels of the 10 novel Prognosis-Associated Genes, Figure S4: Expression correlation between mRNA and miRNA, Table S1: The genes most associated with overall survival (OS) of patients with Stomach adenocarcinoma determined by GEPIA database; Table S2 The genes most associated with disease-free survival (DFS) of patients with stomach adenocarcinoma determined by GEPIA database.

## Abbreviations

Antisense oligonucleotides (ASOs), Carnitine palmitoyltransferase 1C (CPT1C), Circular RNAs (circRNAs), Competing endogenous RNAs (ceRNAs), Complement Factor H-Related Protein 1 (CFHR1), Confidence intervals (CI), Disease-free survival (DFS), ELAV-like RNA-binding protein 3 (ELAVL3), Epstein-Barr virus (EBV), Fatty acid oxidation (FAO), gastric cancer (GC), Genotype-Tissue Expression (GTEx), Hazard ratios (HR), Long non-coding RNAs (lncRNAs), Messenger RNA (mRNA), MicroRNA response elements (MREs), Sodium Leak Channel Non-Selective (NALCN), Overall survival (OS), Stomach adenocarcinoma (STAD), Tigger Transposable Element Derived 6 (TIGD6), Tyrosylprotein Sulfotransferase 1 (TPST1), Zinc Finger Protein 474 (ZNF474).
