# Supplementary material for: A Bioinformatics-Driven ceRNA Network in Stomach Adenocarcinoma: Identification of Novel Prognostic mRNA-miRNA-lncRNA Interactions
Source: Med Sci (Basel). 2025 Oct 1;13(4):214. doi: 10.3390/medsci13040214 (PMC12551037; doi:10.3390/medsci13040214)
Supplement: Supplementary file 1 [file medsci-13-00214-s001.zip › medsci-3863784-supplementary.pdf]

Table S1. The genes most associated with overall survival (OS) of patients with Stomach adenocarcinoma determined by GEPIA database.

Gene Symbol	Gene ID	P-Value
GFAP	ENSG00000131095.11	2.12E-05
RP11-497E19.1	ENSG00000205562.2	2.33E-05
ASPA	ENSG00000108381.10	2.69E-05
SERPINE1	ENSG00000106366.8	3.41E-05
ZNF883	ENSG00000228623.3	3.59E-05
AOC4P	ENSG00000260105.6	3.86E-05
CBLN4	ENSG00000054803.3	3.97E-05
NT5E	ENSG00000135318.11	4.74E-05
AC002480.3	ENSG00000232759.1	6.37E-05
MEI4	ENSG00000269964.2	6.39E-05
CTD-2054N24.2	ENSG00000259363.5	6.93E-05
RP11-1069G10.2	ENSG00000259727.1	9.09E-05
EMX2OS	ENSG00000229847.8	9.24E-05
ZNF192P1	ENSG00000226314.7	9.45E-05
RAI14	ENSG00000039560.13	9.67E-05
TRHDE-AS1	ENSG00000236333.3	1.07E-04
GUCY1A2	ENSG00000152402.10	1.10E-04
PCDHB17P	ENSG00000255622.3	1.12E-04
PLCXD3	ENSG00000182836.9	1.33E-04
RP11-102K13.5	ENSG00000278309.1	1.36E-04
RP11-322E11.5	ENSG00000267583.5	1.40E-04
SORCS3	ENSG00000156395.12	1.75E-04
AKR1B1	ENSG00000085662.13	1.80E-04
CDR1	ENSG00000184258.6	1.90E-04
NRP1	ENSG00000099250.17	2.03E-04
PJA2	ENSG00000198961.9	2.17E-04
SLITRK2	ENSG00000185985.8	2.32E-04
INHBB	ENSG00000163083.5	2.34E-04
LIFR-AS1	ENSG00000244968.6	2.39E-04
CPT1C	ENSG00000169169.14	2.65E-04
GOLGA8IP	ENSG00000277561.4	2.92E-04
SLC52A3	ENSG00000101276.14	3.09E-04
CPNE8	ENSG00000139117.13	3.12E-04
PYGO1	ENSG00000171016.11	3.13E-04
PKNOX2	ENSG00000165495.15	3.29E-04
TFPI2	ENSG00000105825.11	3.39E-04
HTR1F	ENSG00000179097.5	3.43E-04
LINC00315	ENSG00000184274.3	3.53E-04
WDR49	ENSG00000174776.10	3.88E-04
SVEP1	ENSG00000165124.17	3.92E-04
ERBB4	ENSG00000178568.13	3.93E-04
DYNC1I1	ENSG00000158560.14	3.98E-04
ITIH3	ENSG00000162267.12	4.19E-04
ACOT1	ENSG00000184227.7	4.27E-04

MAGED4B	ENSG00000187243.16	4.47E-04
FGF1	ENSG00000113578.17	4.64E-04
COLEC12	ENSG00000158270.11	4.65E-04
CCDC181	ENSG00000117477.12	4.70E-04
DGKQ	ENSG00000145214.13	4.72E-04
EIF3EP1	ENSG00000234882.1	4.84E-04
ATP8A2	ENSG00000132932.16	4.86E-04
PPM1E	ENSG00000175175.5	4.86E-04
CCDC178	ENSG00000166960.16	4.92E-04
AK5	ENSG00000154027.18	4.97E-04
PTPRQ	ENSG00000139304.12	4.98E-04
MUM1L1	ENSG00000157502.12	5.03E-04
BLMH	ENSG00000108578.14	5.04E-04
ANKRD6	ENSG00000135299.16	5.54E-04
PRTG	ENSG00000166450.12	5.68E-04
PCDHB6	ENSG00000113211.5	5.94E-04
BASP1	ENSG00000176788.8	5.95E-04
ASTN1	ENSG00000152092.15	6.06E-04
RP11-469N6.1	ENSG00000251226.1	6.24E-04
C16orf47	ENSG00000197445.2	6.28E-04
VASH2	ENSG00000143494.15	6.30E-04
CTB-31O20.8	ENSG00000267141.1	6.31E-04
AC007228.11	ENSG00000269696.1	6.33E-04
CDC37	ENSG00000105401.6	6.42E-04
GPC3	ENSG00000147257.13	6.44E-04
P4HA3	ENSG00000149380.11	6.45E-04
CASC10	ENSG00000204682.5	6.51E-04
TCEAL7	ENSG00000182916.7	6.90E-04
PDK4	ENSG00000004799.7	7.06E-04
SGCE	ENSG00000127990.15	7.26E-04
MCC	ENSG00000171444.17	7.32E-04
MATN3	ENSG00000132031.12	7.44E-04
RP11-316O14.1	ENSG00000268603.1	7.48E-04
ABCB5	ENSG00000004846.16	7.49E-04
ZNF662	ENSG00000182983.14	7.51E-04
COL4A5	ENSG00000188153.12	7.66E-04
LRFN5	ENSG00000165379.13	7.73E-04
RP11-462L8.1	ENSG00000229656.6	7.89E-04
IQCA1	ENSG00000132321.16	7.95E-04
DPP3	ENSG00000254986.7	7.96E-04
RP11-310P5.1	ENSG00000249650.1	8.09E-04
DSCAM	ENSG00000171587.14	8.13E-04
NPY1R	ENSG00000164128.6	8.14E-04
EGFLAM	ENSG00000164318.17	8.18E-04
FABP4	ENSG00000170323.8	8.20E-04
PCA3	ENSG00000225937.1	8.27E-04
ANKRD53	ENSG00000144031.11	8.36E-04
ADAMTS18	ENSG00000140873.15	8.47E-04
STK32A	ENSG00000169302.14	8.87E-04
CSRNP1	ENSG00000144655.14	9.08E-04

ABCA8	ENSG00000141338.13	9.09E-04
CLRN3	ENSG00000180745.4	9.16E-04
NOVA1	ENSG00000139910.19	9.38E-04
AC005754.8	ENSG00000272108.1	9.43E-04
LINC01436	ENSG00000231106.2	9.50E-04
COMMD10	ENSG00000145781.8	9.50E-04
PCDHB5	ENSG00000113209.8	9.71E-04
PRR15L	ENSG00000167183.2	9.75E-04
ARMCX1	ENSG00000126947.11	9.77E-04
PCOLCE2	ENSG00000163710.7	9.82E-04
NBAT1	ENSG00000260455.1	9.87E-04
ABCA4	ENSG00000198691.11	1.01E-03
SYN2	ENSG00000157152.16	1.01E-03
NAP1L2	ENSG00000186462.8	1.04E-03
SYPL2	ENSG00000143028.8	1.04E-03
RP11-566K19.6	ENSG00000274253.4	1.04E-03
LINC01606	ENSG00000253301.5	1.10E-03
SPX	ENSG00000134548.9	1.10E-03
AC092667.2	ENSG00000230393.1	1.11E-03
CREB5	ENSG00000146592.16	1.11E-03
PGM5P3-AS1	ENSG00000277631.4	1.13E-03
C1GALT1C1L	ENSG00000223658.6	1.14E-03
KCNT2	ENSG00000162687.16	1.14E-03
EMX2	ENSG00000170370.11	1.15E-03
GPNMB	ENSG00000136235.15	1.15E-03
CTD-2525P14.5	ENSG00000280073.1	1.17E-03
RP11-295P9.8	ENSG00000268549.1	1.19E-03
AC005682.6	ENSG00000226329.2	1.19E-03
CD300LG	ENSG00000161649.12	1.20E-03
ANXA8	ENSG00000265190.6	1.21E-03
FREM1	ENSG00000164946.19	1.21E-03
TCEAL5	ENSG00000204065.2	1.27E-03
APOD	ENSG00000189058.8	1.30E-03
TAL2	ENSG00000186051.6	1.32E-03
NLGN4X	ENSG00000146938.14	1.32E-03
GULP1	ENSG00000144366.15	1.33E-03
TRPC6	ENSG00000137672.12	1.34E-03
HOXD1	ENSG00000128645.12	1.35E-03
RP11-731F5.1	ENSG00000254140.1	1.42E-03
CRTAC1	ENSG00000095713.13	1.44E-03
PCDH9	ENSG00000184226.14	1.45E-03
PCDHB4	ENSG00000081818.3	1.47E-03
CDH6	ENSG00000113361.12	1.49E-03
PZP	ENSG00000126838.9	1.50E-03
VCAN	ENSG00000038427.15	1.50E-03
RERG	ENSG00000134533.6	1.54E-03
NALCN	ENSG00000102452.15	1.54E-03
STEAP4	ENSG00000127954.12	1.54E-03
CNRIP1	ENSG00000119865.8	1.54E-03
POU6F2	ENSG00000106536.19	1.56E-03

RP11-60A14.1	ENSG00000279778.1	1.57E-03
LRCOL1	ENSG00000204583.9	1.58E-03
RP3-425C14.4	ENSG00000279453.1	1.58E-03
KCND2	ENSG00000184408.9	1.59E-03
SYT14	ENSG00000143469.16	1.59E-03
AC026904.1	ENSG00000233858.4	1.60E-03
RARB	ENSG00000077092.18	1.61E-03
CYP27C1	ENSG00000186684.12	1.62E-03
RP11-456K23.1	ENSG00000267414.1	1.63E-03
CALCR	ENSG00000004948.13	1.65E-03
DNM1P46	ENSG00000182397.14	1.65E-03
SLC35F1	ENSG00000196376.10	1.70E-03
C1orf95	ENSG00000203685.9	1.71E-03
LINC01537	ENSG00000227467.3	1.73E-03
CADM2	ENSG00000175161.13	1.73E-03
AP000892.6	ENSG00000280143.1	1.74E-03
ITPRIPL1	ENSG00000198885.9	1.74E-03
HOXA10-AS	ENSG00000253187.2	1.77E-03
ACSM5	ENSG00000183549.10	1.77E-03
RBMS1	ENSG00000153250.17	1.81E-03
NOXO1	ENSG00000196408.11	1.81E-03
CPA6	ENSG00000165078.11	1.83E-03
SLC2A3	ENSG00000059804.15	1.85E-03
RAB34	ENSG00000109113.17	1.85E-03
FAM20A	ENSG00000108950.11	1.86E-03
RASSF8	ENSG00000123094.15	1.87E-03
RP11-567J20.2	ENSG00000253688.1	1.87E-03
ZFHX4	ENSG00000091656.15	1.88E-03
MPP4	ENSG00000082126.17	1.89E-03
EIF2AK4	ENSG00000128829.11	1.89E-03
ZNF22	ENSG00000165512.4	1.92E-03
PDLIM1P4	ENSG00000249274.1	1.92E-03
MFAP3L	ENSG00000198948.11	1.92E-03
SRMS	ENSG00000125508.3	1.94E-03
ZNF415P1	ENSG00000266127.1	1.95E-03
TSPAN5	ENSG00000168785.7	1.96E-03
ASF1B	ENSG00000105011.8	1.96E-03
AC091878.1	ENSG00000215196.4	1.97E-03
RP11-211N11.5	ENSG00000234393.1	1.98E-03
NAT8L	ENSG00000185818.7	1.98E-03
NECAB1	ENSG00000123119.11	1.99E-03
ZNF331	ENSG00000130844.16	2.00E-03
FOLR3	ENSG00000110203.8	2.01E-03
EHD3	ENSG00000013016.14	2.01E-03
PTCHD4	ENSG00000244694.7	2.02E-03
BOLA3-AS1	ENSG00000225439.2	2.03E-03
SEC23A	ENSG00000100934.14	2.04E-03
IL1RAPL1	ENSG00000169306.9	2.08E-03
BEX4	ENSG00000102409.9	2.09E-03
AKAP12	ENSG00000131016.16	2.09E-03

NRG3	ENSG00000185737.12	2.10E-03
LINC00961	ENSG00000235387.1	2.11E-03
CLCN3P1	ENSG00000232000.2	2.11E-03
CEP83-AS1	ENSG00000278916.1	2.11E-03
CHAF1A	ENSG00000167670.15	2.11E-03
SCHIP1	ENSG00000151967.18	2.11E-03
OSBPL1A	ENSG00000141447.16	2.14E-03
CHST14	ENSG00000169105.7	2.14E-03
SLC16A7	ENSG00000118596.11	2.17E-03
LINC00989	ENSG00000250334.5	2.28E-03
ACOT2	ENSG00000119673.14	2.30E-03
RP11-620J15.3	ENSG00000257698.1	2.31E-03
EPDR1	ENSG00000086289.11	2.32E-03
RP11-624L4.1	ENSG00000259345.5	2.32E-03
PRL	ENSG00000172179.11	2.36E-03
NTN1	ENSG00000065320.8	2.37E-03
TCF7L1	ENSG00000152284.4	2.38E-03
ITGAV	ENSG00000138448.11	2.41E-03
RP11-59D5__B.2	ENSG00000236345.1	2.41E-03
ZBTB10	ENSG00000205189.11	2.41E-03
GALNT13	ENSG00000144278.14	2.43E-03
CDK15	ENSG00000138395.14	2.44E-03
MIR99AHG	ENSG00000215386.10	2.46E-03
RP11-81K2.2	ENSG00000279036.1	2.47E-03
RP11-64C12.8	ENSG00000267069.1	2.49E-03
LINC00648	ENSG00000259129.5	2.51E-03
LINC00968	ENSG00000246430.6	2.52E-03
GLP2R	ENSG00000065325.12	2.52E-03
WHAMMP3	ENSG00000276141.4	2.53E-03
LGR6	ENSG00000133067.17	2.54E-03
NTAN1	ENSG00000157045.8	2.56E-03
FAM153B	ENSG00000182230.11	2.58E-03
PDE7B	ENSG00000171408.13	2.60E-03
ITGA5	ENSG00000161638.10	2.61E-03
FAM133A	ENSG00000179083.6	2.61E-03
STC1	ENSG00000159167.11	2.63E-03
PHF24	ENSG00000122733.12	2.63E-03
MSC-AS1	ENSG00000235531.9	2.68E-03
RP11-61I13.3	ENSG00000235033.7	2.68E-03
NPAS3	ENSG00000151322.18	2.68E-03
ADRA1D	ENSG00000171873.7	2.68E-03
HAVCR1	ENSG00000113249.12	2.68E-03
AC011239.2	ENSG00000279526.1	2.70E-03
ADGRL4	ENSG00000162618.12	2.74E-03
GOLGA8T	ENSG00000261247.1	2.75E-03
ZNF208	ENSG00000160321.14	2.76E-03
GPX3	ENSG00000211445.11	2.78E-03
LAMA4	ENSG00000112769.18	2.79E-03
CCDC23	ENSG00000177868.11	2.82E-03
AC020571.3	ENSG00000229056.2	2.84E-03

RP11-557L19.1	ENSG00000272002.1	2.84E-03
CACNB4	ENSG00000182389.18	2.84E-03
KIAA1755	ENSG00000149633.11	2.87E-03
RP11-513O13.1	ENSG00000279881.1	2.89E-03
ATP2B3	ENSG00000067842.17	2.89E-03
LOX	ENSG00000113083.12	2.89E-03
ST8SIA6	ENSG00000148488.15	2.90E-03
RP11-180I4.4	ENSG00000268926.2	2.93E-03
RP11-876N24.5	ENSG00000263013.1	2.94E-03
TMEM45A	ENSG00000181458.10	2.95E-03
HIF3A	ENSG00000124440.15	2.97E-03
TUBA3D	ENSG00000075886.10	2.98E-03
CCDC42B	ENSG00000186710.11	2.99E-03
ELAVL3	ENSG00000196361.9	3.01E-03
SHC4	ENSG00000185634.11	3.02E-03
ZNF257	ENSG00000197134.11	3.03E-03
MAGED4	ENSG00000154545.16	3.03E-03
RASSF8-AS1	ENSG00000246695.7	3.06E-03
CELF4	ENSG00000101489.18	3.07E-03
AFF3	ENSG00000144218.18	3.09E-03
GRTP1	ENSG00000139835.13	3.10E-03
BCHE	ENSG00000114200.9	3.14E-03
TPST1	ENSG00000169902.13	3.15E-03
TIGD6	ENSG00000164296.6	3.16E-03
WNK3	ENSG00000196632.10	3.18E-03
C6orf48	ENSG00000204387.12	3.18E-03
CPNE6	ENSG00000100884.9	3.25E-03
TET1	ENSG00000138336.8	3.25E-03
MUSK	ENSG00000030304.12	3.26E-03
PER1	ENSG00000179094.13	3.27E-03
OGN	ENSG00000106809.10	3.29E-03
FBXO17	ENSG00000269190.5	3.30E-03
RP11-401O9.4	ENSG00000273388.1	3.30E-03
AP000476.1	ENSG00000237484.5	3.32E-03
LINC00310	ENSG00000227456.7	3.33E-03
GFRA2	ENSG00000168546.10	3.35E-03
NDUFAF3	ENSG00000178057.14	3.35E-03
CTD-2536I1.2	ENSG00000280304.1	3.39E-03
MOSPD1	ENSG00000101928.12	3.45E-03
PLAT	ENSG00000104368.17	3.46E-03
LINC00619	ENSG00000204187.5	3.46E-03
C19orf26	ENSG00000099625.12	3.49E-03
IGFBP7-AS1	ENSG00000245067.6	3.52E-03
ZNF300	ENSG00000145908.12	3.54E-03
RP11-553A21.3	ENSG00000231652.2	3.56E-03
MDP1	ENSG00000213920.8	3.58E-03
NKAIN2	ENSG00000188580.13	3.59E-03
ARAP1-AS2	ENSG00000245148.2	3.59E-03
MOGAT1	ENSG00000124003.12	3.60E-03
AL592528.1	ENSG00000205424.1	3.64E-03

EFNA3	ENSG00000143590.13	3.65E-03
GPR173	ENSG00000184194.5	3.68E-03
GABARAPL2	ENSG00000034713.7	3.70E-03
G0S2	ENSG00000123689.5	3.70E-03
SV2B	ENSG00000185518.11	3.73E-03
RGS4	ENSG00000117152.13	3.75E-03
KCNIP1	ENSG00000182132.12	3.77E-03
RAMP1	ENSG00000132329.10	3.78E-03
THRB	ENSG00000151090.17	3.84E-03
AP001626.2	ENSG00000235023.1	3.86E-03
AFAP1L1	ENSG00000157510.13	3.86E-03
MAGI2-AS3	ENSG00000234456.7	3.86E-03
AC016995.3	ENSG00000231367.5	3.88E-03
LRAT	ENSG00000121207.11	3.88E-03
RP11-681H18.2	ENSG00000277801.1	3.90E-03
RP13-514E23.1	ENSG00000261496.1	3.93E-03
PCDHB16	ENSG00000272674.3	3.95E-03
DOCK4	ENSG00000128512.19	3.97E-03
CDO1	ENSG00000129596.4	3.99E-03
RP6-109B7.5	ENSG00000273289.1	3.99E-03
KIAA1324L	ENSG00000164659.14	3.99E-03
USP51	ENSG00000247746.4	3.99E-03
CTD-2008L17.2	ENSG00000206129.3	4.00E-03
AC004947.2	ENSG00000233760.1	4.03E-03
DZIP1	ENSG00000134874.17	4.05E-03
TCN2	ENSG00000185339.8	4.06E-03
RP11-713P17.3	ENSG00000204241.7	4.08E-03
PTPN6	ENSG00000111679.16	4.11E-03
IGFBP7	ENSG00000163453.11	4.11E-03
AC114730.2	ENSG00000235151.1	4.12E-03
RP11-542B15.1	ENSG00000203585.3	4.12E-03
CCNA1	ENSG00000133101.9	4.12E-03
GPR156	ENSG00000175697.10	4.12E-03
RP11-632L2.2	ENSG00000278177.1	4.15E-03
MFGE8	ENSG00000140545.14	4.18E-03
IGHE	ENSG00000211891.5	4.20E-03
RP11-108M12.3	ENSG00000258592.1	4.22E-03
VIPAS39	ENSG00000151445.15	4.23E-03
HGF	ENSG00000019991.15	4.25E-03
SPIRE1	ENSG00000134278.14	4.25E-03
ZNF229	ENSG00000278318.4	4.26E-03
ZNF423	ENSG00000102935.11	4.27E-03
RP11-274H2.3	ENSG00000240032.1	4.29E-03
CERS5	ENSG00000139624.12	4.30E-03
NACAD	ENSG00000136274.8	4.31E-03
BICC1	ENSG00000122870.11	4.33E-03
AP4S1	ENSG00000100478.14	4.33E-03
GTF2H2C	ENSG00000183474.15	4.34E-03
PCDHB3	ENSG00000113205.4	4.35E-03
EYA1	ENSG00000104313.17	4.38E-03

RP11-600F24.1	ENSG00000243904.1	4.38E-03
THBS1	ENSG00000137801.10	4.39E-03
QKI	ENSG00000112531.16	4.43E-03
PHKG1	ENSG00000164776.9	4.45E-03
CHRFAM7A	ENSG00000166664.13	4.48E-03
MTTP	ENSG00000138823.12	4.50E-03
CD109	ENSG00000156535.13	4.52E-03
PRSS3	ENSG00000010438.16	4.53E-03
DPT	ENSG00000143196.4	4.56E-03
EDNRB	ENSG00000136160.14	4.58E-03
KLHDC2	ENSG00000165516.10	4.58E-03
HAGLR	ENSG00000224189.6	4.60E-03
MSRB3	ENSG00000174099.10	4.61E-03
RP11-398K22.12	ENSG00000229852.2	4.62E-03
ARMCX2	ENSG00000184867.13	4.62E-03
NAP1L6	ENSG00000204118.1	4.63E-03
CSMD2	ENSG00000121904.17	4.64E-03
DIRC1	ENSG00000174325.4	4.66E-03
TREML4	ENSG00000188056.11	4.69E-03
ACVR1	ENSG00000115170.13	4.69E-03
APBB1	ENSG00000166313.18	4.71E-03
ANO4	ENSG00000151572.16	4.72E-03
COL24A1	ENSG00000171502.14	4.72E-03
C1QTNF4	ENSG00000172247.3	4.73E-03
CAND2	ENSG00000144712.11	4.73E-03
EEF1A2	ENSG00000101210.10	4.74E-03
AK4	ENSG00000162433.14	4.75E-03
WDR17	ENSG00000150627.15	4.76E-03
LINC00475	ENSG00000225511.6	4.78E-03
AC009948.5	ENSG00000223960.6	4.79E-03
RP11-155D18.13	ENSG00000280422.1	4.79E-03
CLIP3	ENSG00000105270.14	4.80E-03
PCDHB13	ENSG00000187372.11	4.80E-03
AC000403.4	ENSG00000278727.1	4.82E-03
RP3-388E23.2	ENSG00000234084.1	4.82E-03
NTMT1	ENSG00000148335.14	4.85E-03
ADRA1B	ENSG00000170214.3	4.85E-03
RAB19	ENSG00000146955.10	4.85E-03
CRB1	ENSG00000134376.14	4.85E-03
ZNF474	ENSG00000164185.4	4.87E-03
GPR176	ENSG00000166073.8	4.87E-03
PSD4	ENSG00000125637.15	4.87E-03
LGALS12	ENSG00000133317.14	4.88E-03
RPL21P40	ENSG00000235670.1	4.89E-03
MYL3	ENSG00000160808.9	4.92E-03
NPTX1	ENSG00000171246.5	4.95E-03
LINC01140	ENSG00000267272.5	4.98E-03
NUDT10	ENSG00000122824.10	5.01E-03
POT1-AS1	ENSG00000224897.6	5.01E-03
AC002456.2	ENSG00000223969.5	5.05E-03

PCCA	ENSG00000175198.14	5.07E-03
UBE2QL1	ENSG00000215218.3	5.08E-03
C6orf120	ENSG00000185127.6	5.08E-03
GLT8D1	ENSG00000016864.16	5.10E-03
DIMT1	ENSG00000086189.9	5.10E-03
CHRD	ENSG00000090539.15	5.10E-03
ZFPM2	ENSG00000169946.13	5.13E-03
TGFB2	ENSG00000092969.11	5.14E-03
C5	ENSG00000106804.7	5.16E-03
DMRTC1B	ENSG00000184911.14	5.16E-03
CAV1	ENSG00000105974.11	5.17E-03
TNFAIP8L3	ENSG00000183578.5	5.19E-03
HSPD1P11	ENSG00000251348.1	5.19E-03
EBF2	ENSG00000221818.8	5.21E-03
GJA1	ENSG00000152661.7	5.21E-03
C1QTNF2	ENSG00000145861.7	5.22E-03
PCDHA1	ENSG00000204970.9	5.22E-03
CTB-134H23.3	ENSG00000260908.1	5.23E-03
FLJ16779	ENSG00000275620.1	5.23E-03
LINC00922	ENSG00000261742.5	5.24E-03
ARGLU1	ENSG00000134884.13	5.24E-03
MASP1	ENSG00000127241.16	5.25E-03
SOCS2	ENSG00000120833.13	5.29E-03
SOX7	ENSG00000171056.7	5.31E-03
CITED2	ENSG00000164442.9	5.31E-03
NREP	ENSG00000134986.13	5.32E-03
SLITRK4	ENSG00000179542.15	5.34E-03
RNF217	ENSG00000146373.16	5.34E-03
WASF1	ENSG00000112290.12	5.36E-03
SYT6	ENSG00000134207.14	5.40E-03
MMP19	ENSG00000123342.15	5.41E-03
PTPRD	ENSG00000153707.15	5.44E-03
RP11-311D14.1	ENSG00000248778.1	5.46E-03
ILDR1	ENSG00000145103.12	5.46E-03
REEP4	ENSG00000168476.11	5.49E-03
RP4-545K15.5	ENSG00000261101.2	5.49E-03
ZRANB2-AS2	ENSG00000229956.9	5.51E-03
IL34	ENSG00000157368.10	5.52E-03
WBSCR17	ENSG00000185274.11	5.52E-03
SV2A	ENSG00000159164.9	5.52E-03
KCNK2	ENSG00000082482.13	5.58E-03
SIAH3	ENSG00000215475.4	5.60E-03
RP11-114H24.2	ENSG00000260776.5	5.61E-03
FAM181B	ENSG00000182103.4	5.62E-03
PPP1R14A	ENSG00000167641.10	5.64E-03
NAMA	ENSG00000271086.5	5.66E-03
IL1R1	ENSG00000115594.11	5.69E-03
CCNDBP1	ENSG00000166946.13	5.69E-03
RAB9B	ENSG00000123570.3	5.70E-03
PDGFD	ENSG00000170962.12	5.71E-03

ZC3H12C	ENSG00000149289.10	5.71E-03
APOC3	ENSG00000110245.11	5.71E-03
RP11-284F21.7	ENSG00000229953.1	5.72E-03
UBE2Q2	ENSG00000140367.11	5.75E-03
STARD9	ENSG00000159433.11	5.75E-03
NT5C1A	ENSG00000116981.3	5.77E-03
CFHR1	ENSG00000244414.6	5.77E-03
PROSER2-AS1	ENSG00000225778.5	5.78E-03
IL1RL1	ENSG00000115602.16	5.78E-03
RP11-594N15.3	ENSG00000260398.1	5.82E-03
BRINP1	ENSG00000078725.12	5.83E-03
CCNO	ENSG00000152669.8	5.83E-03
SYT4	ENSG00000132872.11	5.85E-03
LMOD3	ENSG00000163380.15	5.85E-03
AR	ENSG00000169083.15	5.85E-03
SCG2	ENSG00000171951.4	5.86E-03
PIM1	ENSG00000137193.13	5.87E-03
MAPK4	ENSG00000141639.11	5.89E-03
RAB12	ENSG00000206418.3	5.89E-03
TMEM200B	ENSG00000253304.1	5.89E-03
LCN6	ENSG00000267206.5	5.94E-03
VGLL3	ENSG00000206538.7	5.99E-03
AL022393.9	ENSG00000280107.1	5.99E-03
BRMS1L	ENSG00000100916.13	6.00E-03
RP11-368L12.1	ENSG00000260658.5	6.01E-03
FNDC1	ENSG00000164694.16	6.03E-03
RP11-433J20.1	ENSG00000236990.1	6.03E-03
DDIT4L	ENSG00000145358.6	6.03E-03
RP11-490O6.2	ENSG00000262420.3	6.05E-03
ZBTB16	ENSG00000109906.13	6.06E-03
THSD7A	ENSG00000005108.15	6.12E-03
LINC00856	ENSG00000230417.10	6.13E-03
TECTA	ENSG00000109927.9	6.14E-03
RP11-221N13.3	ENSG00000256268.1	6.19E-03
TAL1	ENSG00000162367.11	6.19E-03
C6	ENSG00000039537.13	6.24E-03
LINC00997	ENSG00000281332.1	6.26E-03
CTD-2033D15.2	ENSG00000276107.1	6.28E-03
LIPM	ENSG00000173239.13	6.29E-03
MYOZ3	ENSG00000164591.13	6.33E-03
ISLR2	ENSG00000167178.15	6.35E-03
GFRA3	ENSG00000146013.10	6.35E-03
VIPR2	ENSG00000106018.13	6.35E-03
RECK	ENSG00000122707.11	6.36E-03
RP11-286H14.8	ENSG00000243230.1	6.37E-03
SOCS3	ENSG00000184557.4	6.38E-03
ELANE	ENSG00000197561.6	6.38E-03
PITX2	ENSG00000164093.15	6.39E-03
AC124944.3	ENSG00000226155.1	6.39E-03
COLEC11	ENSG00000118004.17	6.44E-03

RET	ENSG00000165731.17	6.45E-03
RGS5	ENSG00000143248.12	6.45E-03
SNCA	ENSG00000145335.15	6.46E-03
IGSF21	ENSG00000117154.11	6.46E-03
DIRC3	ENSG00000231672.6	6.48E-03
RP11-136C24.3	ENSG00000273291.5	6.50E-03

Table S2. The genes most associated with disease-free survival (DFS) of patients with stomach adenocarcinoma determined by GEPIA database.

Gene Symbol	Gene ID	P-Value
LINC01529	ENSG00000225872.2	8.03E-07
ANXA8	ENSG00000265190.6	1.12E-06
GPR87	ENSG00000138271.5	4.82E-06
ALOX12P2	ENSG00000262943.7	8.29E-06
AC009542.2	ENSG00000231794.5	1.05E-05
WNT7A	ENSG00000154764.5	1.75E-05
SORCS3	ENSG00000156395.12	2.73E-05
RP3-439F8.1	ENSG00000234869.1	2.75E-05
CTD-2591A1.1	ENSG00000280159.1	2.76E-05
NTRK3	ENSG00000140538.16	2.78E-05
ITIH3	ENSG00000162267.12	2.81E-05
TCEAL5	ENSG00000204065.2	3.15E-05
ATP5F1P5	ENSG00000254944.1	3.17E-05
AC002480.3	ENSG00000232759.1	3.44E-05
SLC35F3	ENSG00000183780.12	3.49E-05
MUSK	ENSG00000030304.12	4.55E-05
TEKT2	ENSG00000092850.11	4.56E-05
POU1F1	ENSG00000064835.10	4.99E-05
MYOZ3	ENSG00000164591.13	5.00E-05
CTB-134H23.3	ENSG00000260908.1	5.30E-05
CTD-2114J12.1	ENSG00000253525.1	5.99E-05
C7orf57	ENSG00000164746.13	6.02E-05
RN7SKP296	ENSG00000223117.1	6.03E-05
ABCA6	ENSG00000154262.12	6.19E-05
RP11-430H10.1	ENSG00000254427.1	6.20E-05
RP11-759A24.1	ENSG00000279645.1	6.31E-05
CGB5	ENSG00000189052.6	6.53E-05
NKAIN4	ENSG00000101198.14	6.66E-05
RP11-169F17.1	ENSG00000263711.5	7.73E-05
RP11-401O9.4	ENSG00000273388.1	8.45E-05
ANKRD6	ENSG00000135299.16	8.48E-05
NTF4	ENSG00000225950.7	9.44E-05
BNC1	ENSG00000169594.12	9.75E-05
MAGI2	ENSG00000187391.17	1.10E-04
TCL6	ENSG00000187621.14	1.15E-04
ELANE	ENSG00000197561.6	1.18E-04
LRR1	ENSG00000165501.16	1.21E-04
CCDC178	ENSG00000166960.16	1.22E-04
CCNA1	ENSG00000133101.9	1.22E-04

BLK	ENSG00000136573.12	1.28E-04
LINC00648	ENSG00000259129.5	1.30E-04
LTBP3	ENSG00000168056.14	1.31E-04
GULP1	ENSG00000144366.15	1.35E-04
COL6A4P1	ENSG00000230524.8	1.37E-04
MAB21L1	ENSG00000180660.7	1.40E-04
TMEM132C	ENSG00000181234.9	1.44E-04
POT1-AS1	ENSG00000224897.6	1.45E-04
RP11-613F22.8	ENSG00000256588.1	1.48E-04
PNPT1P1	ENSG00000229241.1	1.49E-04
MIR99AHG	ENSG00000215386.10	1.52E-04
COL4A3	ENSG00000169031.18	1.54E-04
TECTA	ENSG00000109927.9	1.55E-04
GEMIN8	ENSG00000046647.13	1.57E-04
AC005753.1	ENSG00000278925.1	1.67E-04
RP11-278J6.4	ENSG00000279130.1	1.68E-04
UPK1B	ENSG00000114638.7	1.69E-04
EFNA5	ENSG00000184349.12	1.71E-04
DMRTA2	ENSG00000142700.11	1.75E-04
VTN	ENSG00000109072.13	1.77E-04
CACNB4	ENSG00000182389.18	1.77E-04
C9orf152	ENSG00000188959.9	1.78E-04
C19orf84	ENSG00000262874.1	1.84E-04
WNT7B	ENSG00000188064.9	1.87E-04
CDK15	ENSG00000138395.14	1.92E-04
RP11-449J21.5	ENSG00000267128.1	1.93E-04
TMEM132E	ENSG00000181291.6	1.96E-04
RP11-327J17.9	ENSG00000277135.1	1.98E-04
TRIM54	ENSG00000138100.13	1.98E-04
RP11-713P17.3	ENSG00000204241.7	1.99E-04
ACSM6	ENSG00000173124.14	1.99E-04
TOB2P1	ENSG00000176933.5	1.99E-04
AKAP12	ENSG00000131016.16	2.05E-04
TTTY14	ENSG00000176728.7	2.38E-04
PCDHAC1	ENSG00000248383.4	2.42E-04
NPY1R	ENSG00000164128.6	2.50E-04
RP11-620J15.3	ENSG00000257698.1	2.55E-04
LINC01152	ENSG00000256124.5	2.56E-04
GFRA3	ENSG00000146013.10	2.64E-04
MRPS2	ENSG00000122140.10	2.72E-04
ACADL	ENSG00000115361.7	2.81E-04
NR2F2-AS1	ENSG00000247809.7	2.85E-04
TF	ENSG00000091513.14	2.88E-04
RP11-834C11.6	ENSG00000249388.1	2.90E-04
RP11-349H17.2	ENSG00000275805.1	2.93E-04
EBF4	ENSG00000088881.20	2.95E-04
NAA10	ENSG00000102030.15	2.96E-04
TMEM37	ENSG00000171227.6	2.99E-04
CYP8B1	ENSG00000180432.5	3.02E-04
KCNN1	ENSG00000105642.15	3.03E-04

CYP46A1	ENSG00000036530.8	3.03E-04
CCDC152	ENSG00000198865.9	3.04E-04
RP11-59D5__B.2	ENSG00000236345.1	3.04E-04
GPR15	ENSG00000154165.4	3.07E-04
SV2A	ENSG00000159164.9	3.09E-04
DAPK1	ENSG00000196730.12	3.16E-04
GCGR	ENSG00000215644.9	3.21E-04
APBB1	ENSG00000166313.18	3.26E-04
GRPEL1	ENSG00000109519.12	3.29E-04
LINC00565	ENSG00000260910.1	3.33E-04
CYP24A1	ENSG00000019186.9	3.35E-04
CTD-3157E16.1	ENSG00000265519.1	3.37E-04
PCDHA1	ENSG00000204970.9	3.39E-04
RP4-803A2.1	ENSG00000217644.5	3.41E-04
TUB	ENSG00000166402.8	3.46E-04
P2RX2	ENSG00000187848.12	3.49E-04
CTD-3010D24.3	ENSG00000263893.2	3.50E-04
GRID1	ENSG00000182771.17	3.50E-04
AC007319.1	ENSG00000224063.5	3.55E-04
MPRIP-AS1	ENSG00000225442.2	3.55E-04
LINC01426	ENSG00000234380.1	3.59E-04
DKK1	ENSG00000107984.9	3.80E-04
C10orf10	ENSG00000165507.8	3.80E-04
RP6-74O6.6	ENSG00000272824.1	3.82E-04
PACS1	ENSG00000175115.11	3.92E-04
RP4-806M20.4	ENSG00000268649.3	3.95E-04
RP11-482D24.3	ENSG00000257918.1	3.95E-04
GLIS2	ENSG00000126603.8	4.05E-04
PCDH15	ENSG00000150275.17	4.07E-04
MRPL35	ENSG00000132313.14	4.07E-04
FLOT2	ENSG00000132589.15	4.16E-04
STK32A	ENSG00000169302.14	4.19E-04
AC006460.2	ENSG00000228509.5	4.19E-04
CYP11A1	ENSG00000140459.17	4.23E-04
PCOLCE2	ENSG00000163710.7	4.23E-04
CTD-2015G9.2	ENSG00000261175.5	4.31E-04
RP11-60A14.1	ENSG00000279778.1	4.42E-04
ARRDC3	ENSG00000113369.8	4.46E-04
ARHGAP31-AS1	ENSG00000241155.1	4.53E-04
LINC00652	ENSG00000179935.9	4.54E-04
RP11-433P17.3	ENSG00000262118.1	4.58E-04
CTD-2311M21.3	ENSG00000261821.2	4.61E-04
RP11-181K12.1	ENSG00000279481.1	4.61E-04
RP11-44D5.1	ENSG00000270409.1	4.62E-04
CLDN6	ENSG00000184697.6	4.68E-04
TRHDE-AS1	ENSG00000236333.3	4.68E-04
AP000662.4	ENSG00000254602.1	4.79E-04
RP11-63M22.1	ENSG00000260558.1	4.89E-04
KCNS1	ENSG00000124134.8	4.91E-04
NOVA1	ENSG00000139910.19	4.91E-04

CLDN11	ENSG00000013297.10	4.94E-04
TCEAL7	ENSG00000182916.7	4.98E-04
CSMD1	ENSG00000183117.17	4.99E-04
TGM5	ENSG00000104055.14	5.03E-04
CKS2	ENSG00000123975.4	5.08E-04
RP11-981P6.1	ENSG00000258302.2	5.13E-04
TRPC1	ENSG00000144935.14	5.20E-04
PCDHB4	ENSG00000081818.3	5.31E-04
CH17-360D5.2	ENSG00000276850.4	5.36E-04
AIFM1	ENSG00000156709.13	5.41E-04
LINC00506	ENSG00000281392.1	5.41E-04
ABCA8	ENSG00000141338.13	5.48E-04
RP11-435O5.2	ENSG00000237857.2	5.52E-04
CTB-134H23.2	ENSG00000196796.5	5.58E-04
CFHR1	ENSG00000244414.6	5.59E-04
C9orf40	ENSG00000135045.6	5.66E-04
WNT1	ENSG00000125084.11	5.68E-04
SLC30A8	ENSG00000164756.12	5.73E-04
RARB	ENSG00000077092.18	5.81E-04
FAM174B	ENSG00000185442.12	5.84E-04
CPEB1	ENSG00000214575.9	5.98E-04
RP11-102K13.5	ENSG00000278309.1	6.03E-04
ST6GAL2	ENSG00000144057.15	6.20E-04
TPST1	ENSG00000169902.13	6.21E-04
IRX6	ENSG00000159387.7	6.28E-04
LINC00346	ENSG00000255874.2	6.46E-04
FLJ16779	ENSG00000275620.1	6.51E-04
RPL23AP58	ENSG00000228657.1	6.53E-04
AP001626.2	ENSG00000235023.1	6.57E-04
KCNT2	ENSG00000162687.16	6.57E-04
KLHL38	ENSG00000175946.8	6.75E-04
AR	ENSG00000169083.15	6.83E-04
THSD4	ENSG00000187720.14	6.87E-04
FGF14	ENSG00000102466.15	6.95E-04
SCN5A	ENSG00000183873.15	7.09E-04
RIMS4	ENSG00000101098.12	7.10E-04
PCDHB5	ENSG00000113209.8	7.17E-04
ARMCX4	ENSG00000196440.11	7.17E-04
C17orf50	ENSG00000270806.1	7.25E-04
PTPRQ	ENSG00000139304.12	7.32E-04
CAPG	ENSG00000042493.15	7.33E-04
PRTG	ENSG00000166450.12	7.35E-04
SLCO4A1	ENSG00000101187.15	7.40E-04
UBXN7-AS1	ENSG00000225822.4	7.42E-04
RP11-209D14.4	ENSG00000266126.1	7.49E-04
C12orf77	ENSG00000226397.7	7.53E-04
HERC2P5	ENSG00000260644.6	7.55E-04
RP11-445P17.8	ENSG00000224034.1	7.61E-04
IGHD	ENSG00000211898.7	7.66E-04
RP11-1260E13.4	ENSG00000262061.5	7.72E-04

ZDHHC2	ENSG00000104219.12	7.73E-04
NDUFA8	ENSG00000119421.6	7.80E-04
RP11-1191J2.5	ENSG00000272927.1	7.92E-04
WDR91	ENSG00000105875.13	7.94E-04
LINC00540	ENSG00000276476.2	8.03E-04
LURAP1	ENSG00000171357.5	8.13E-04
RP11-94C24.13	ENSG00000275897.1	8.36E-04
PALM	ENSG00000099864.17	8.37E-04
FBXO27	ENSG00000161243.8	8.42E-04
RP11-80I15.1	ENSG00000223849.1	8.51E-04
RP11-697N18.1	ENSG00000251354.3	8.71E-04
SLC4A5	ENSG00000188687.15	8.73E-04
RP11-80A15.1	ENSG00000258744.1	8.75E-04
RP11-474C8.8	ENSG00000274124.1	8.82E-04
RP11-497E19.1	ENSG00000205562.2	8.85E-04
AC004538.3	ENSG00000230333.6	8.92E-04
DYNLRB2	ENSG00000168589.14	9.21E-04
RP1-29C18.8	ENSG00000235111.1	9.27E-04
CTD-2555C10.3	ENSG00000259230.1	9.27E-04
CTC-487M23.7	ENSG00000272389.1	9.28E-04
SMURF1	ENSG00000198742.9	9.35E-04
SH3GL3	ENSG00000140600.16	9.35E-04
FAM216B	ENSG00000179813.6	9.45E-04
GJA1P1	ENSG00000176857.5	9.46E-04
SIGLEC6	ENSG00000105492.15	9.47E-04
HSPB1P2	ENSG00000230216.1	9.48E-04
PLCXD3	ENSG00000182836.9	9.51E-04
TTC21B-AS1	ENSG00000224490.5	9.53E-04
LSAMP-AS1	ENSG00000240922.1	9.56E-04
ITIH4	ENSG00000055955.15	9.67E-04
YPEL4	ENSG00000166793.10	9.70E-04
CLEC4F	ENSG00000152672.7	9.77E-04
CALCR	ENSG00000004948.13	9.78E-04
RP5-1139B12.2	ENSG00000269890.1	9.80E-04
RP11-823P9.4	ENSG00000279107.1	9.87E-04
AL591893.1	ENSG00000229021.2	9.90E-04
PCDHA13	ENSG00000239389.7	9.93E-04
SCARA5	ENSG00000168079.16	9.94E-04
VWA5B1	ENSG00000158816.15	9.94E-04
RP11-890B15.2	ENSG00000254842.6	9.95E-04
PCDHAC2	ENSG00000243232.4	1.01E-03
TP53TG3D	ENSG00000205456.11	1.01E-03
RP11-522B15.3	ENSG00000259275.2	1.04E-03
RP11-161I6.2	ENSG00000263745.5	1.04E-03
RFPL1S	ENSG00000225465.8	1.04E-03
C1QTNF4	ENSG00000172247.3	1.05E-03
ANKRD53	ENSG00000144031.11	1.05E-03
CTSG	ENSG00000100448.3	1.06E-03
RP11-399B17.1	ENSG00000278962.1	1.06E-03
RP11-353N14.2	ENSG00000262772.1	1.08E-03

AC019117.2	ENSG00000236039.1	1.08E-03
PCDHB12	ENSG00000120328.6	1.08E-03
C9orf41	ENSG00000156017.12	1.08E-03
RAB40A	ENSG00000172476.3	1.10E-03
RP11-483C6.1	ENSG00000262119.1	1.10E-03
RTL1	ENSG00000254656.1	1.11E-03
PCNA	ENSG00000132646.10	1.11E-03
SLC45A1	ENSG00000162426.14	1.11E-03
RP5-1185I7.1	ENSG00000232756.1	1.11E-03
HNRNPA1P12	ENSG00000220157.4	1.12E-03
NALCN	ENSG00000102452.15	1.13E-03
LINC01260	ENSG00000132832.9	1.13E-03
FAM198A	ENSG00000144649.8	1.14E-03
RP11-306O13.1	ENSG00000213121.2	1.15E-03
RPP25L	ENSG00000164967.9	1.15E-03
RP11-307C18.1	ENSG00000272950.1	1.17E-03
SYPL2	ENSG00000143028.8	1.18E-03
LRAT	ENSG00000121207.11	1.18E-03
RP11-81H3.2	ENSG00000251138.6	1.18E-03
HAVCR1	ENSG00000113249.12	1.18E-03
LRRTM1	ENSG00000162951.10	1.19E-03
LRFN5	ENSG00000165379.13	1.19E-03
RP11-542B15.1	ENSG00000203585.3	1.20E-03
LINC00473	ENSG00000223414.2	1.22E-03
RP1-302G2.5	ENSG00000262179.2	1.24E-03
FAM153B	ENSG00000182230.11	1.24E-03
ONECUT1	ENSG00000169856.8	1.25E-03
RBP4	ENSG00000138207.12	1.25E-03
RP11-209M4.1	ENSG00000267253.1	1.25E-03
EGOT	ENSG00000235947.1	1.26E-03
L3MBTL3	ENSG00000198945.7	1.27E-03
MMACHC	ENSG00000132763.14	1.27E-03
DRC7	ENSG00000159625.14	1.27E-03
NPTX1	ENSG00000171246.5	1.28E-03
WTAPP1	ENSG00000255282.6	1.28E-03
GRIK4	ENSG00000149403.11	1.30E-03
GNAS-AS1	ENSG00000235590.7	1.31E-03
AC004813.1	ENSG00000279777.1	1.32E-03
TCP11	ENSG00000124678.17	1.32E-03
RP11-806O11.1	ENSG00000253671.1	1.32E-03
OR5K2	ENSG00000231861.2	1.33E-03
RP11-631M6.3	ENSG00000251682.1	1.34E-03
RNF183	ENSG00000165188.13	1.35E-03
RERG	ENSG00000134533.6	1.38E-03
SH3GL2	ENSG00000107295.9	1.40E-03
CLRN3	ENSG00000180745.4	1.40E-03
DCLK1	ENSG00000133083.14	1.41E-03
ZNF667	ENSG00000198046.11	1.41E-03
CTD-2215E18.1	ENSG00000251513.2	1.42E-03
SCTR	ENSG00000080293.9	1.43E-03

PRSS50	ENSG00000206549.12	1.44E-03
AP001626.1	ENSG00000225431.1	1.44E-03
KPNA7	ENSG00000185467.7	1.45E-03
RP11-712P20.2	ENSG00000266965.1	1.46E-03
ZNF474	ENSG00000164185.4	1.46E-03
ENPP1	ENSG00000197594.11	1.46E-03
RP11-48B3.3	ENSG00000254162.1	1.47E-03
RP11-295M18.6	ENSG00000272823.1	1.47E-03
CTD-2536I1.2	ENSG00000280304.1	1.47E-03
KIRREL-IT1	ENSG00000226520.1	1.48E-03
FLJ45079	ENSG00000204283.3	1.48E-03
FAM153C	ENSG00000204677.10	1.48E-03
CNTN2	ENSG00000184144.9	1.50E-03
AC013275.2	ENSG00000231013.1	1.52E-03
SLC15A2	ENSG00000163406.10	1.53E-03
UNC13C	ENSG00000137766.16	1.54E-03
PCDHA3	ENSG00000255408.3	1.54E-03
FGF19	ENSG00000162344.3	1.57E-03
SLC5A10	ENSG00000154025.15	1.59E-03
CD300LG	ENSG00000161649.12	1.59E-03
RP11-454L9.2	ENSG00000259318.1	1.59E-03
RPL21P40	ENSG00000235670.1	1.60E-03
TBC1D27	ENSG00000128438.10	1.61E-03
AC092162.1	ENSG00000230552.5	1.61E-03
RP11-284F21.10	ENSG00000272405.1	1.62E-03
ASCL1	ENSG00000139352.3	1.62E-03
SEC14L3	ENSG00000100012.11	1.64E-03
KLB	ENSG00000134962.6	1.64E-03
BACH1-AS1	ENSG00000232118.2	1.64E-03
LA16c-3G11.7	ENSG00000241838.3	1.65E-03
RP11-474O21.5	ENSG00000272482.1	1.65E-03
RP11-438L7.3	ENSG00000255967.1	1.67E-03
KIF5A	ENSG00000155980.11	1.67E-03
ZSCAN10	ENSG00000130182.7	1.70E-03
FKBP4P6	ENSG00000268234.1	1.71E-03
RP11-616M22.11	ENSG00000273551.1	1.71E-03
AC011997.1	ENSG00000222017.1	1.72E-03
RP11-789C17.1	ENSG00000265413.1	1.72E-03
BEST3	ENSG00000127325.18	1.74E-03
SUMO4	ENSG00000177688.6	1.76E-03
RP11-37N22.1	ENSG00000214803.3	1.78E-03
ARAP1-AS2	ENSG00000245148.2	1.78E-03
RP11-462L8.1	ENSG00000229656.6	1.79E-03
RP11-586K12.1	ENSG00000279795.1	1.79E-03
CYP27C1	ENSG00000186684.12	1.80E-03
RAB34	ENSG00000109113.17	1.80E-03
MNS1	ENSG00000138587.5	1.81E-03
KRT79	ENSG00000185640.5	1.81E-03
PI3	ENSG00000124102.4	1.82E-03
FAM193B	ENSG00000146067.15	1.82E-03

LRTM2	ENSG00000166159.10	1.83E-03
CDC42P5	ENSG00000253439.1	1.83E-03
CFAP221	ENSG00000163075.12	1.84E-03
RP11-266K4.14	ENSG00000275367.1	1.85E-03
RP4-565E6.1	ENSG00000227733.8	1.85E-03
SVEP1	ENSG00000165124.17	1.85E-03
SYN1	ENSG00000008056.12	1.86E-03
XXyac-YX155B6.5	ENSG00000232265.7	1.88E-03
OGN	ENSG00000106809.10	1.90E-03
ITFG2	ENSG00000111203.11	1.90E-03
GALNT16	ENSG00000100626.16	1.92E-03
SUSD5	ENSG00000173705.8	1.92E-03
CTD-2195B23.3	ENSG00000269652.1	1.92E-03
ZNF367	ENSG00000165244.6	1.93E-03
PP2D1	ENSG00000183977.13	1.93E-03
RPL7AP65	ENSG00000228000.1	1.94E-03
RNF219-AS1	ENSG00000234377.7	1.94E-03
NIPAL4	ENSG00000172548.14	1.94E-03
TVP23A	ENSG00000166676.14	1.96E-03
HNRNPA1P8	ENSG00000229251.3	1.97E-03
CTD-2530N21.4	ENSG00000254064.1	1.98E-03
HCAR1	ENSG00000196917.5	1.98E-03
INHBA-AS1	ENSG00000224116.6	1.98E-03
RP11-266K4.9	ENSG00000215241.3	1.99E-03
A2M-AS1	ENSG00000245105.2	2.02E-03
NUDT2	ENSG00000164978.17	2.02E-03
NIM1K	ENSG00000177453.7	2.02E-03
RNF165	ENSG00000141622.13	2.02E-03
HCG4B	ENSG00000227262.3	2.02E-03
EFCAB12	ENSG00000172771.11	2.03E-03
AC108676.1	ENSG00000244675.2	2.03E-03
RN7SL683P	ENSG00000242330.3	2.03E-03
SPIRE1	ENSG00000134278.14	2.04E-03
NTNG1	ENSG00000162631.18	2.04E-03
JAZF1-AS1	ENSG00000234336.6	2.06E-03
FGF14-AS2	ENSG00000272143.1	2.06E-03
RP11-23D24.2	ENSG00000238755.3	2.07E-03
NBAT1	ENSG00000260455.1	2.08E-03
SP7	ENSG00000170374.5	2.08E-03
CPNE8	ENSG00000139117.13	2.08E-03
AC093642.1	ENSG00000280119.1	2.08E-03
SERPINA4	ENSG00000100665.11	2.08E-03
LINC00202-2	ENSG00000231976.7	2.11E-03
NPAS3	ENSG00000151322.18	2.12E-03
MAB21L2	ENSG00000181541.5	2.13E-03
WNT10B	ENSG00000169884.13	2.13E-03
RP11-303E16.6	ENSG00000261838.5	2.13E-03
RPL10P1	ENSG00000217026.3	2.15E-03
TBX6	ENSG00000149922.10	2.17E-03
CPT1C	ENSG00000169169.14	2.18E-03

KIRREL2	ENSG00000126259.19	2.18E-03
PZP	ENSG00000126838.9	2.19E-03
FOXE1	ENSG00000178919.8	2.19E-03
OCA2	ENSG00000104044.15	2.21E-03
RPL23AP23	ENSG00000236863.2	2.21E-03
KIF21B	ENSG00000116852.14	2.22E-03
DMRTC1B	ENSG00000184911.14	2.22E-03
SCRN1	ENSG00000136193.16	2.23E-03
OPN3	ENSG00000054277.12	2.23E-03
USE1	ENSG00000053501.12	2.23E-03
NBPF14	ENSG00000270629.5	2.23E-03
PPP4R1L	ENSG00000124224.16	2.26E-03
NUDT7	ENSG00000140876.11	2.28E-03
IGSF10	ENSG00000152580.8	2.29E-03
RP11-110I1.14	ENSG00000271751.1	2.30E-03
RP11-382B18.1	ENSG00000279417.1	2.30E-03
TSNAXIP1	ENSG00000102904.14	2.31E-03
RP11-243M5.2	ENSG00000280200.1	2.31E-03
ONECUT2	ENSG00000119547.5	2.32E-03
UG0898H09	ENSG00000274956.2	2.33E-03
RP11-463O9.9	ENSG00000270020.1	2.33E-03
TSPY26P	ENSG00000235217.6	2.34E-03
ZBTB20-AS1	ENSG00000241560.5	2.34E-03
ZFPM2	ENSG00000169946.13	2.35E-03
LINC01176	ENSG00000281404.1	2.35E-03
TBC1D3B	ENSG00000274808.4	2.36E-03
RP11-867G23.10	ENSG00000254510.1	2.36E-03
KLHL14	ENSG00000197705.9	2.36E-03
RP11-470L19.2	ENSG00000235407.1	2.36E-03
TET1	ENSG00000138336.8	2.38E-03
MAN2B1	ENSG00000104774.12	2.38E-03
MAP2	ENSG00000078018.19	2.39E-03
RP4-724E13.2	ENSG00000228204.2	2.40E-03
CLTA	ENSG00000122705.16	2.40E-03
CTC-480C2.1	ENSG00000250874.1	2.40E-03
CFH	ENSG00000000971.15	2.41E-03
RP11-426C22.4	ENSG00000259807.1	2.41E-03
GBA2	ENSG00000070610.14	2.41E-03
PTPN5	ENSG00000110786.17	2.41E-03
POLR3DP1	ENSG00000214626.2	2.42E-03
CTF1	ENSG00000150281.6	2.42E-03
SIGLEC17P	ENSG00000171101.13	2.43E-03
STXBP6	ENSG00000168952.15	2.43E-03
GDF6	ENSG00000156466.9	2.43E-03
CCNDBP1	ENSG00000166946.13	2.43E-03
TNNT3	ENSG00000130595.16	2.43E-03
RP11-123C21.2	ENSG00000270986.1	2.44E-03
CTD-3035K23.3	ENSG00000279713.1	2.45E-03
Mar-10	ENSG00000173838.11	2.46E-03
COMMD10	ENSG00000145781.8	2.47E-03

ELAVL3	ENSG00000196361.9	2.47E-03
CDC20B	ENSG00000164287.12	2.47E-03
NDRG4	ENSG00000103034.14	2.48E-03
TMEM240	ENSG00000205090.8	2.49E-03
SDCCAG3P2	ENSG00000181101.7	2.49E-03
CCDC181	ENSG00000117477.12	2.49E-03
SERPINA5	ENSG00000188488.13	2.49E-03
TIGD6	ENSG00000164296.6	2.49E-03
XXbac-BPG154L12.4	ENSG00000225914.1	2.49E-03
FTO-IT1	ENSG00000260936.1	2.50E-03
RP11-73M18.6	ENSG00000270108.1	2.52E-03
ZMAT2	ENSG00000146007.10	2.54E-03
ABCA9-AS1	ENSG00000231749.3	2.55E-03
ABHD8	ENSG00000127220.5	2.56E-03
RP4-665N4.4	ENSG00000232862.5	2.56E-03
ISCA1	ENSG00000135070.13	2.56E-03
RPL5P17	ENSG00000243859.3	2.57E-03
RP11-297J22.1	ENSG00000271709.1	2.58E-03
LOXL4	ENSG00000138131.3	2.59E-03
RP11-544L8__B.4	ENSG00000175967.3	2.60E-03
GS1-120K12.4	ENSG00000260976.1	2.62E-03
RP11-1000B6.7	ENSG00000276724.1	2.63E-03
KCNS2	ENSG00000156486.7	2.63E-03
BIRC7	ENSG00000101197.12	2.65E-03
LRRC3B	ENSG00000179796.11	2.65E-03
NOX5	ENSG00000255346.9	2.66E-03
TSPYL2	ENSG00000184205.14	2.67E-03
CTB-33G10.1	ENSG00000243829.1	2.67E-03
FAM231D	ENSG00000272058.2	2.68E-03
TTTY15	ENSG00000233864.7	2.69E-03
CASC10	ENSG00000204682.5	2.70E-03
ASF1B	ENSG00000105011.8	2.70E-03
HMGB1P23	ENSG00000253770.1	2.71E-03
RP11-894J14.2	ENSG00000279144.1	2.71E-03
SLC18A2	ENSG00000165646.11	2.72E-03
ZNF677	ENSG00000197928.10	2.72E-03
RP11-64B16.2	ENSG00000213144.2	2.73E-03
PNMA1	ENSG00000176903.4	2.74E-03
CTD-2509G16.2	ENSG00000255002.1	2.74E-03
ZNF660	ENSG00000144792.9	2.74E-03
PCDHB18P	ENSG00000146001.5	2.74E-03
APOA1-AS	ENSG00000235910.1	2.74E-03
ZNF790-AS1	ENSG00000267254.5	2.75E-03
MIR181A1HG	ENSG00000229989.3	2.75E-03
BCAS3	ENSG00000141376.20	2.75E-03
FAT2	ENSG00000086570.12	2.77E-03
TUBA4B	ENSG00000243910.7	2.78E-03
PRKG1-AS1	ENSG00000236671.7	2.78E-03
RP11-148O21.2	ENSG00000255354.1	2.78E-03

CASC18	ENSG00000257859.1	2.79E-03
CDHR4	ENSG00000187492.8	2.79E-03
RPS15AP38	ENSG00000237668.1	2.81E-03
CASP5	ENSG00000137757.10	2.81E-03
ERBB4	ENSG00000178568.13	2.83E-03
UNC13D	ENSG00000092929.11	2.83E-03
RP11-936I5.1	ENSG00000266998.1	2.84E-03
BCL2L10	ENSG00000137875.4	2.84E-03
MGAT5B	ENSG00000167889.12	2.85E-03
DTX1	ENSG00000135144.7	2.85E-03
NECAP1P2	ENSG00000234632.1	2.86E-03
RP11-615I2.2	ENSG00000260577.2	2.87E-03

CALCR

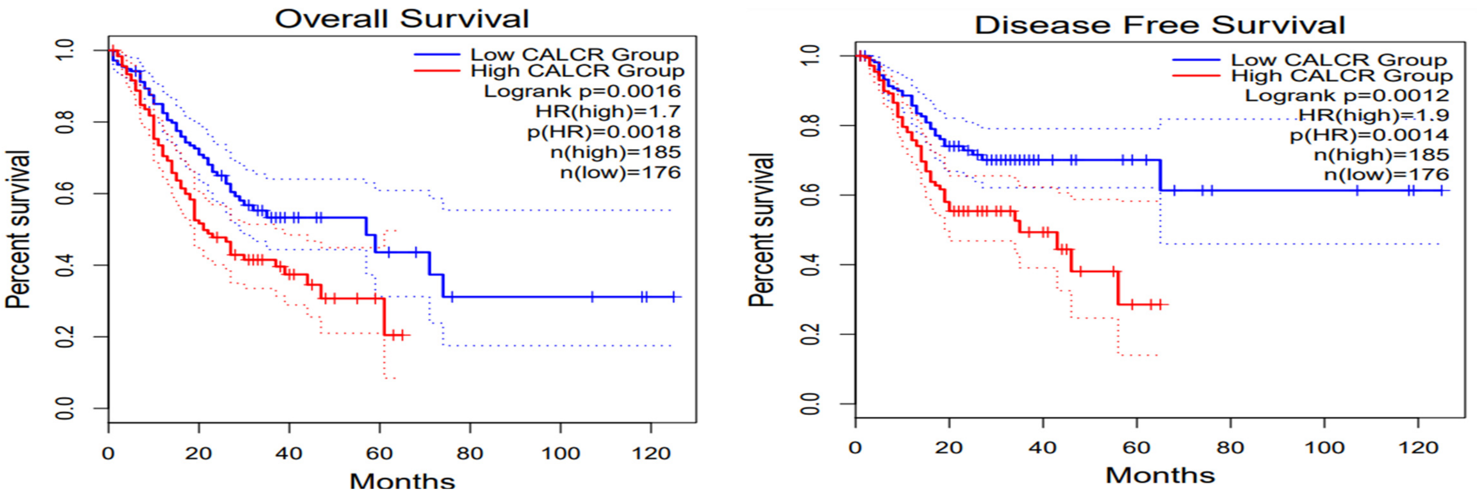

CFHR1

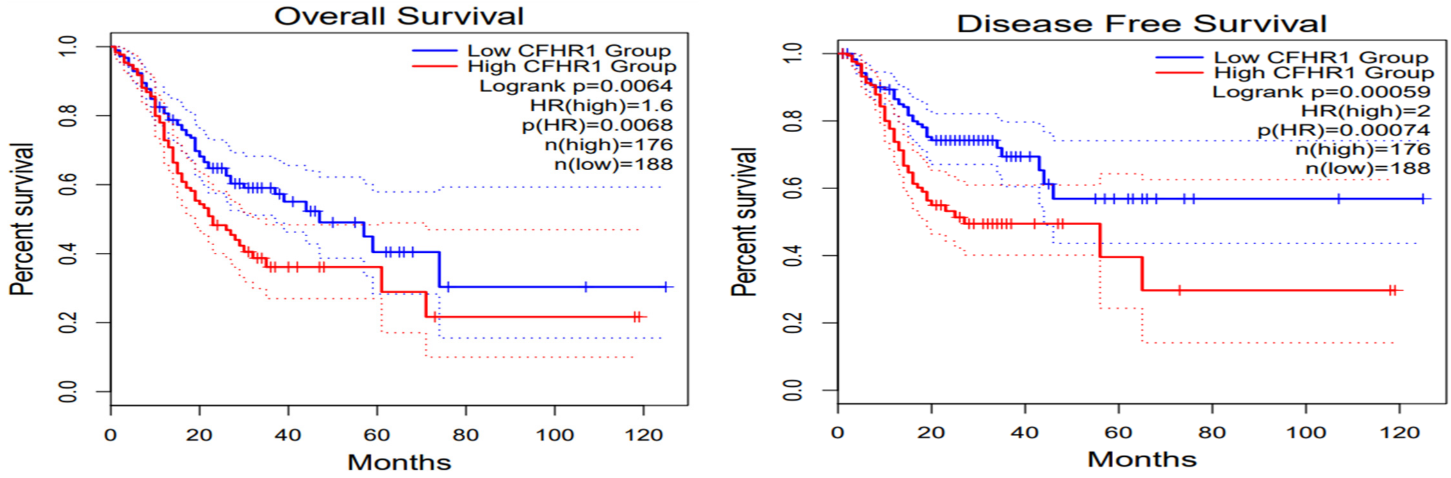

CPT1C

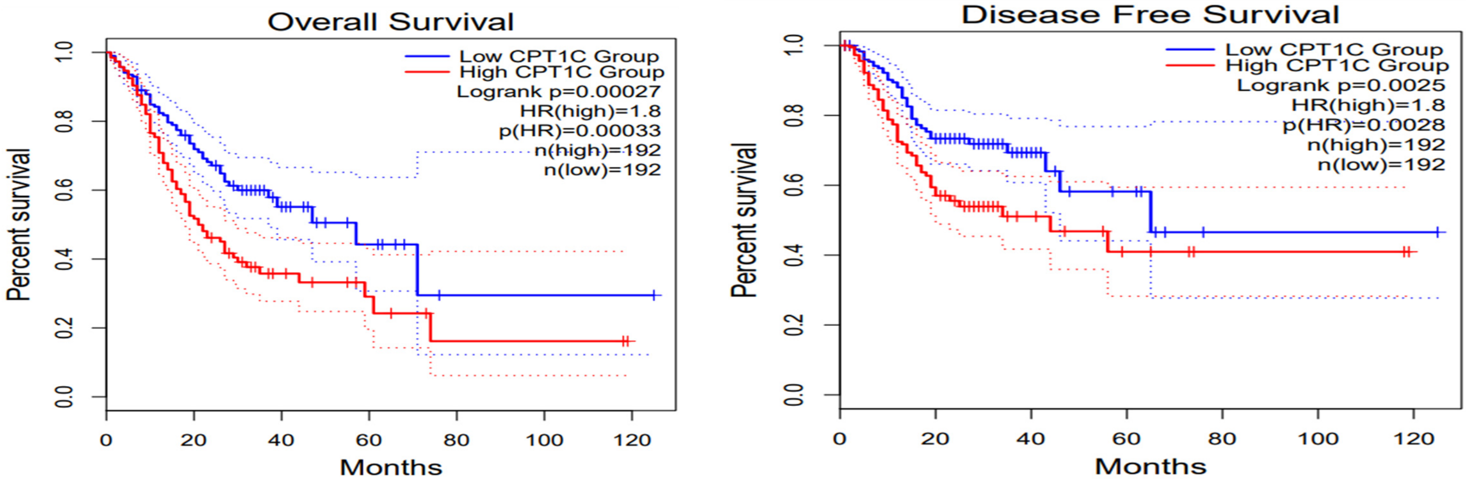

ELAVL3

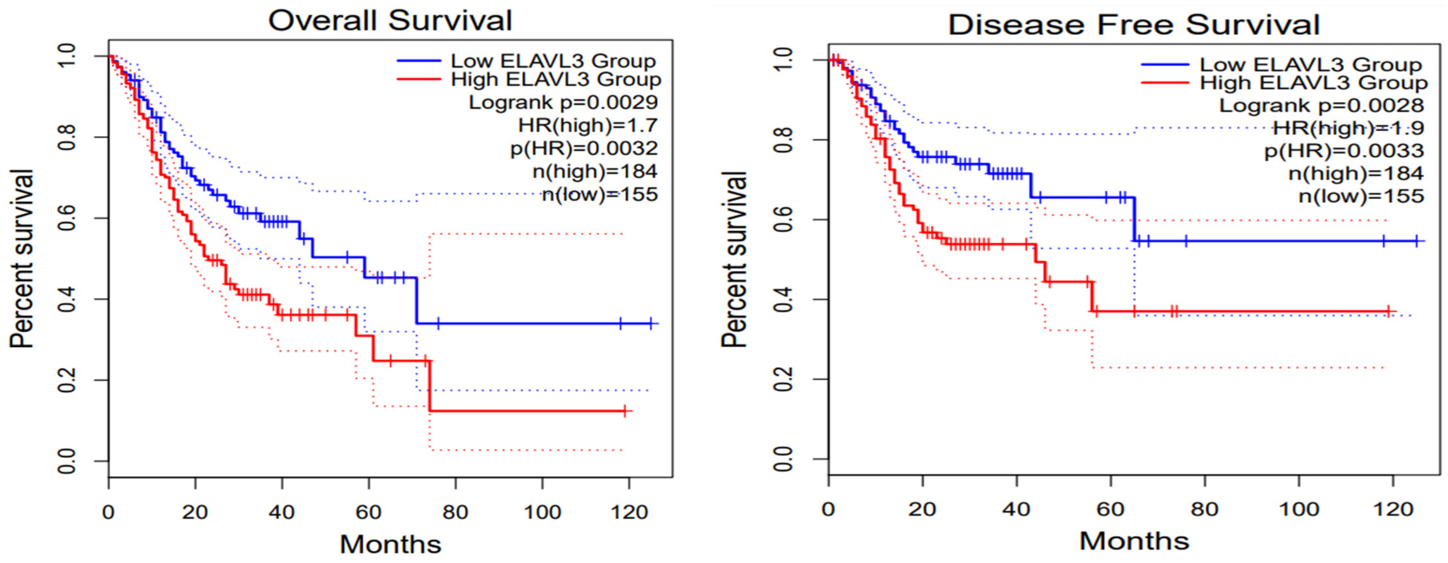

FLJ16779

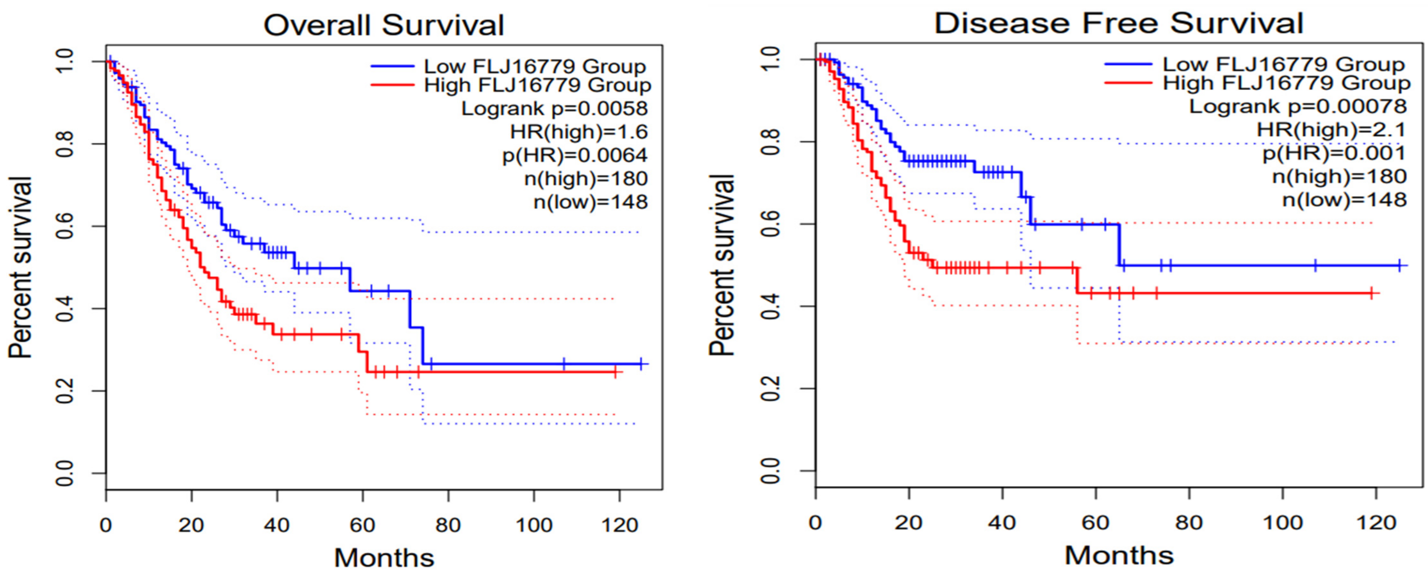

MYOZ3

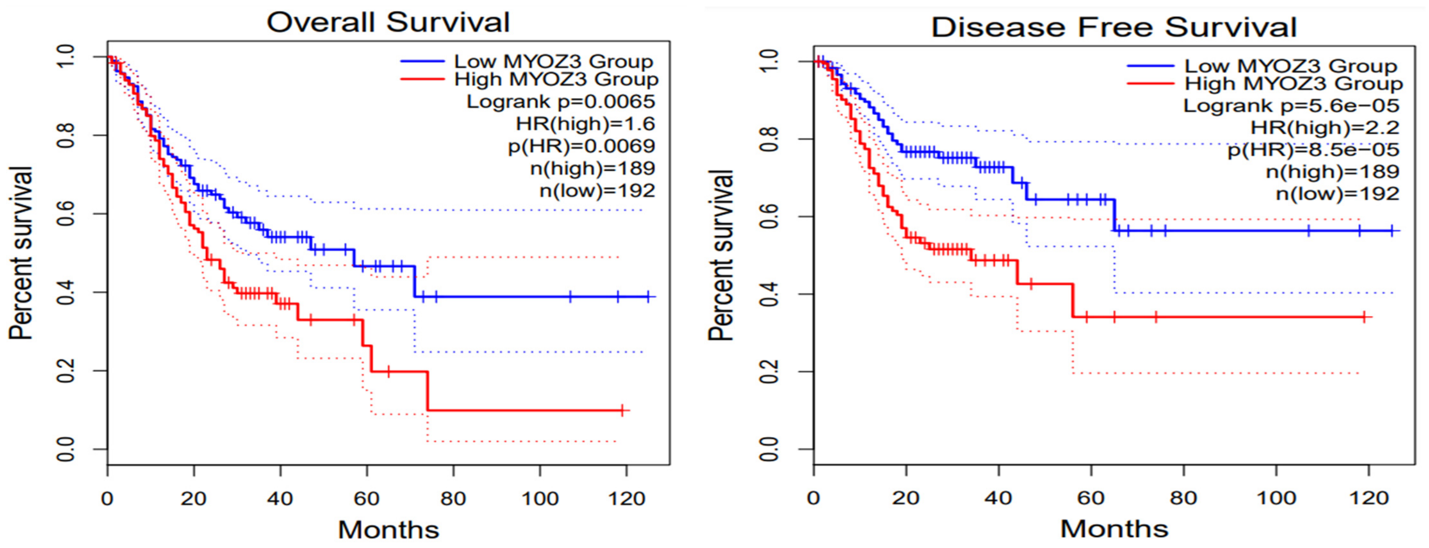

NALCN

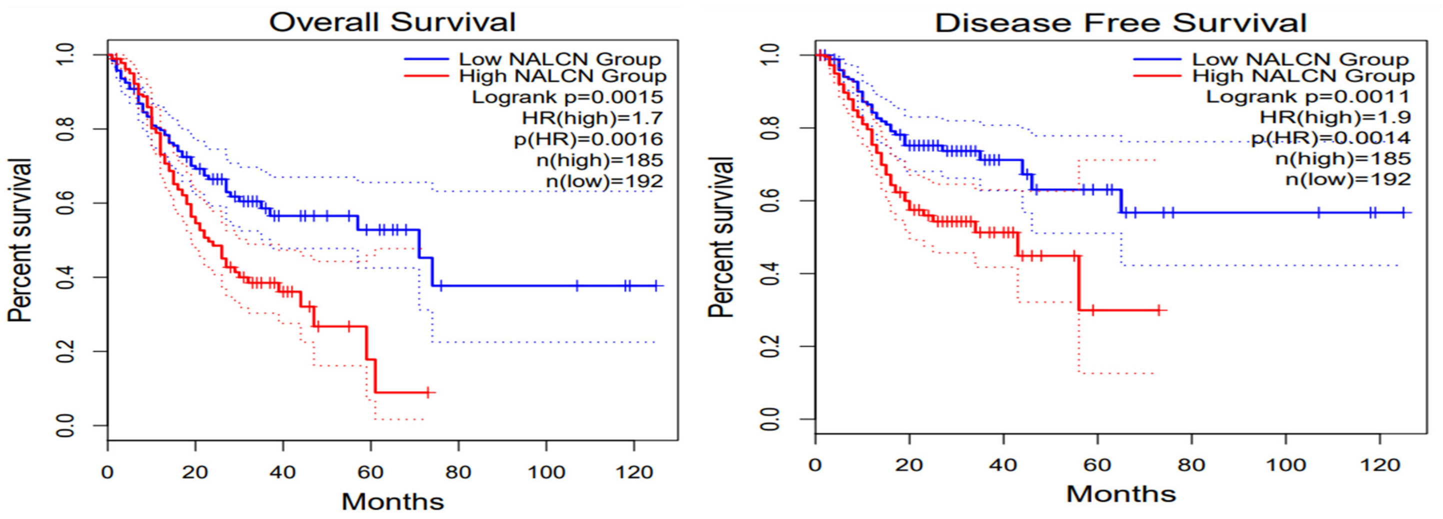

TIGD6

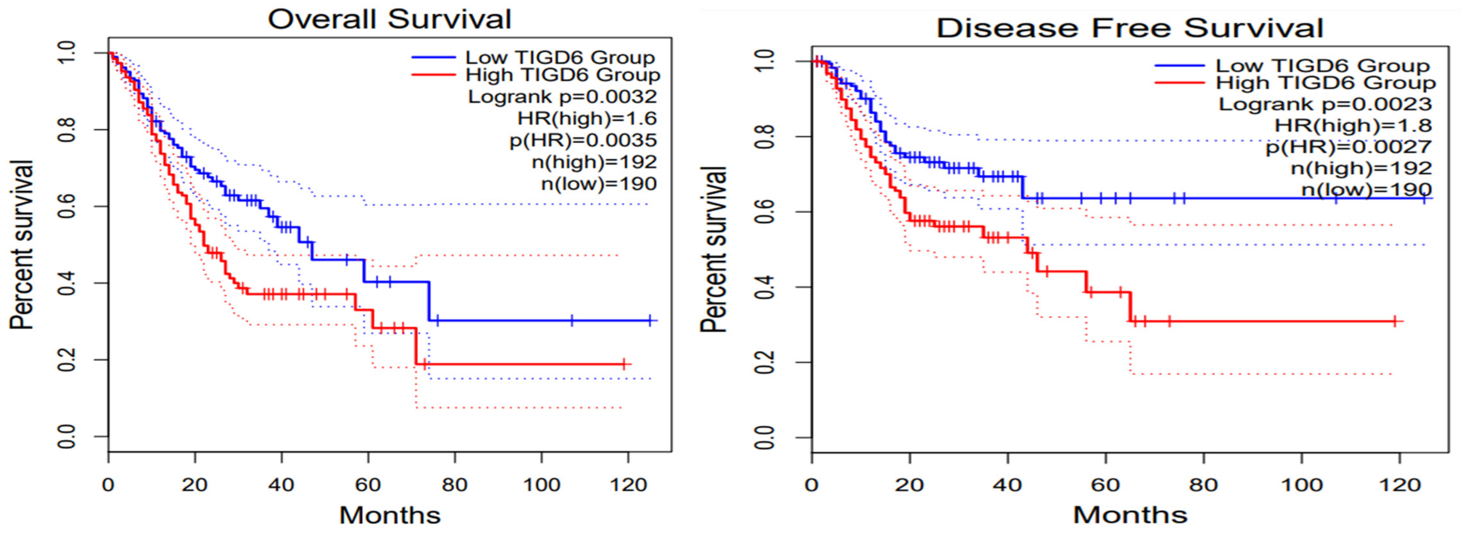

TPST1

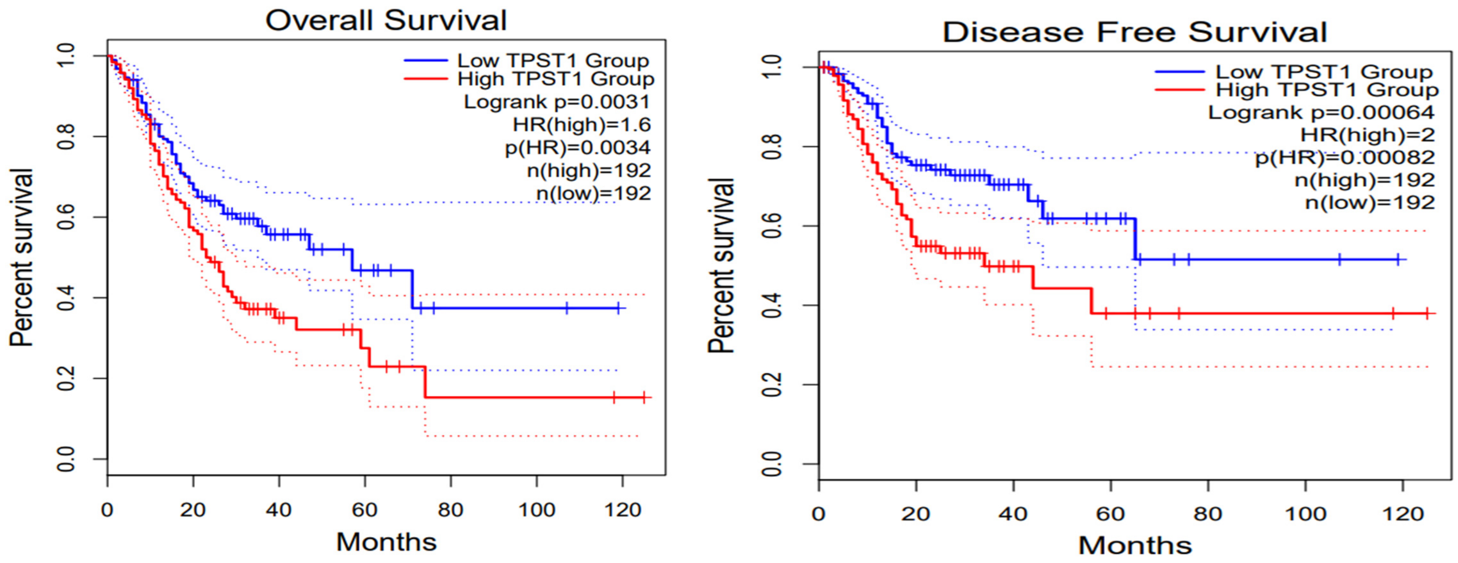

ZNF474

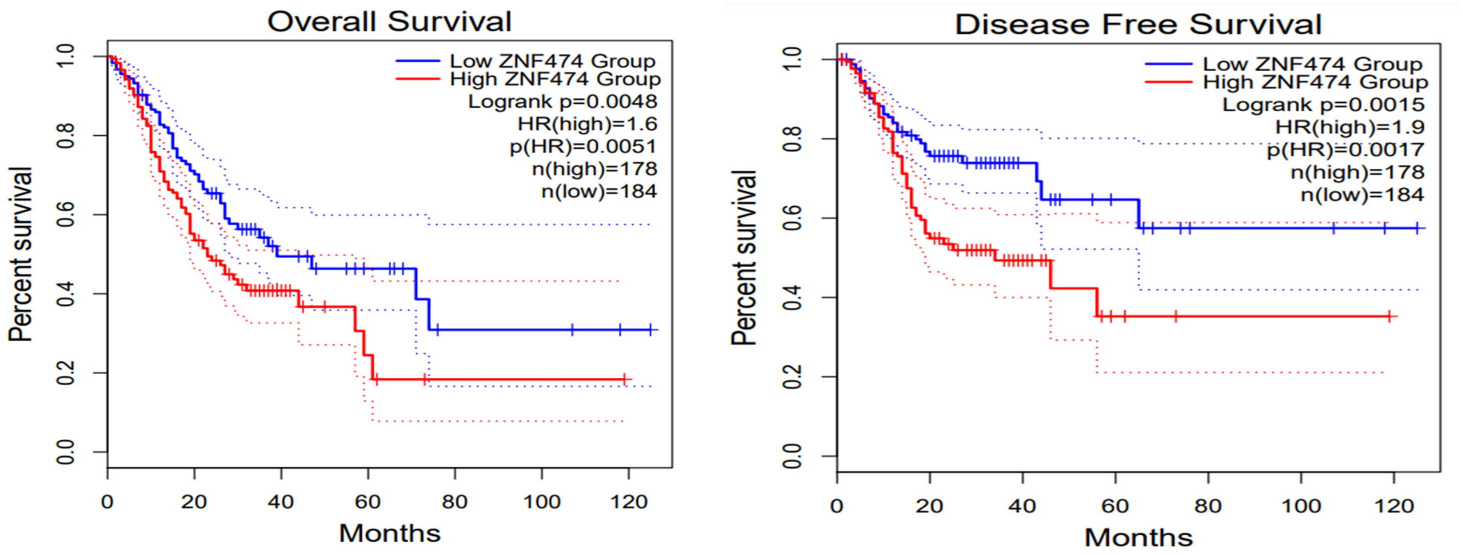

Figure S1. Expression of the 10 novel genes in stomach adenocarcinoma and their prognostic value, using GEPIA database.

High expression of the 10 novel genes is significantly associated with poor overall survival and disease-free survival in stomach adenocarcinoma.

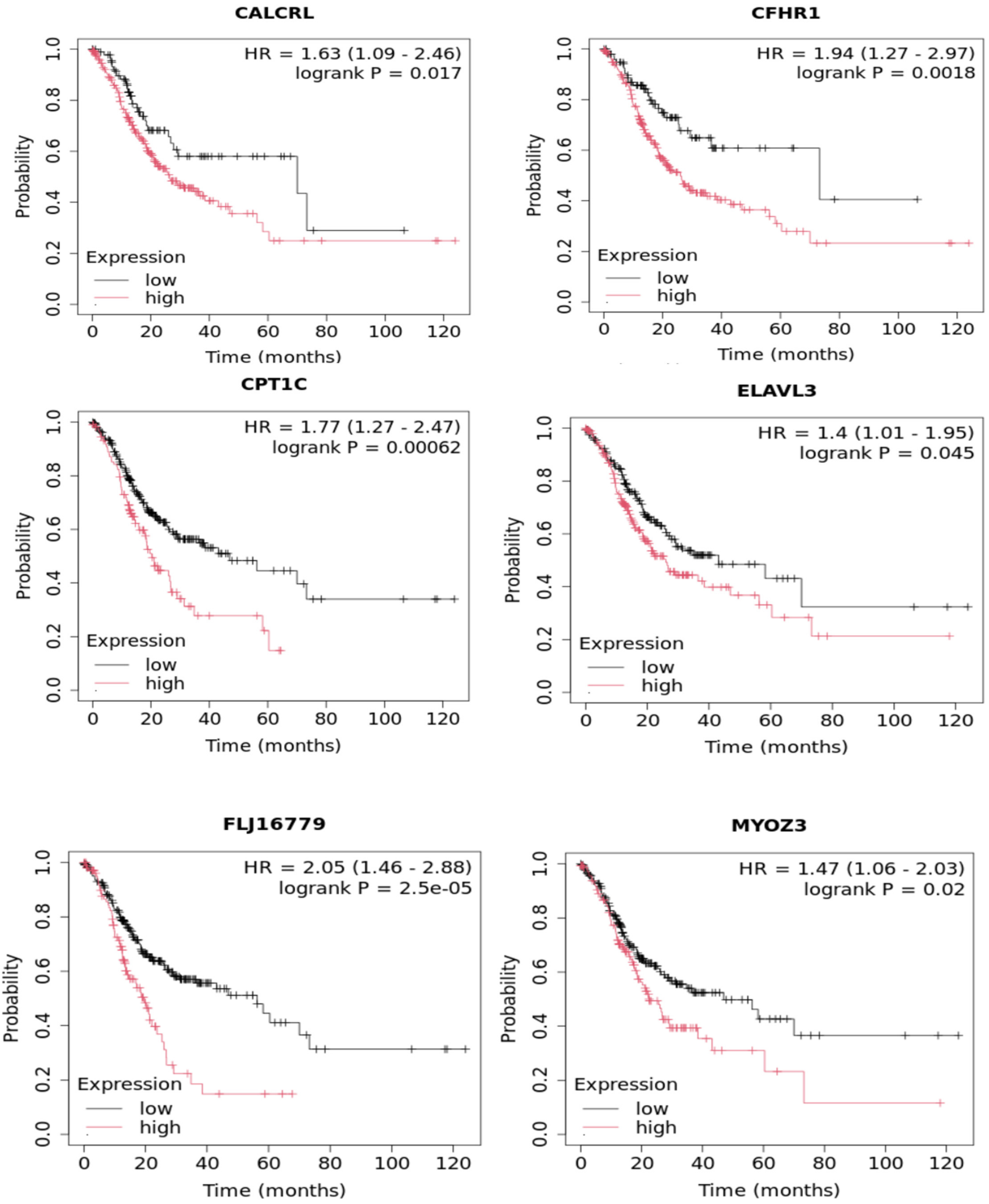
CALCRL
HR = 1.63 (1.09 - 2.46)
logrank P = 0.017
Probability
Time (months)
Expression
low
high
CFHR1
HR = 1.94 (1.27 - 2.97)
logrank P = 0.0018
Probability
Time (months)
Expression
low
high
CPT1C
HR = 1.77 (1.27 - 2.47)
logrank P = 0.00062
Probability
Time (months)
Expression
low
high
ELAVL3
HR = 1.4 (1.01 - 1.95)
logrank P = 0.045
Probability
Time (months)
Expression
low
high
FLJ16779
HR = 2.05 (1.46 - 2.88)
logrank P = 2.5e-05
Probability
Time (months)
Expression
low
high
MYOZ3
HR = 1.47 (1.06 - 2.03)
logrank P = 0.02
Probability
Time (months)
Expression
low
high

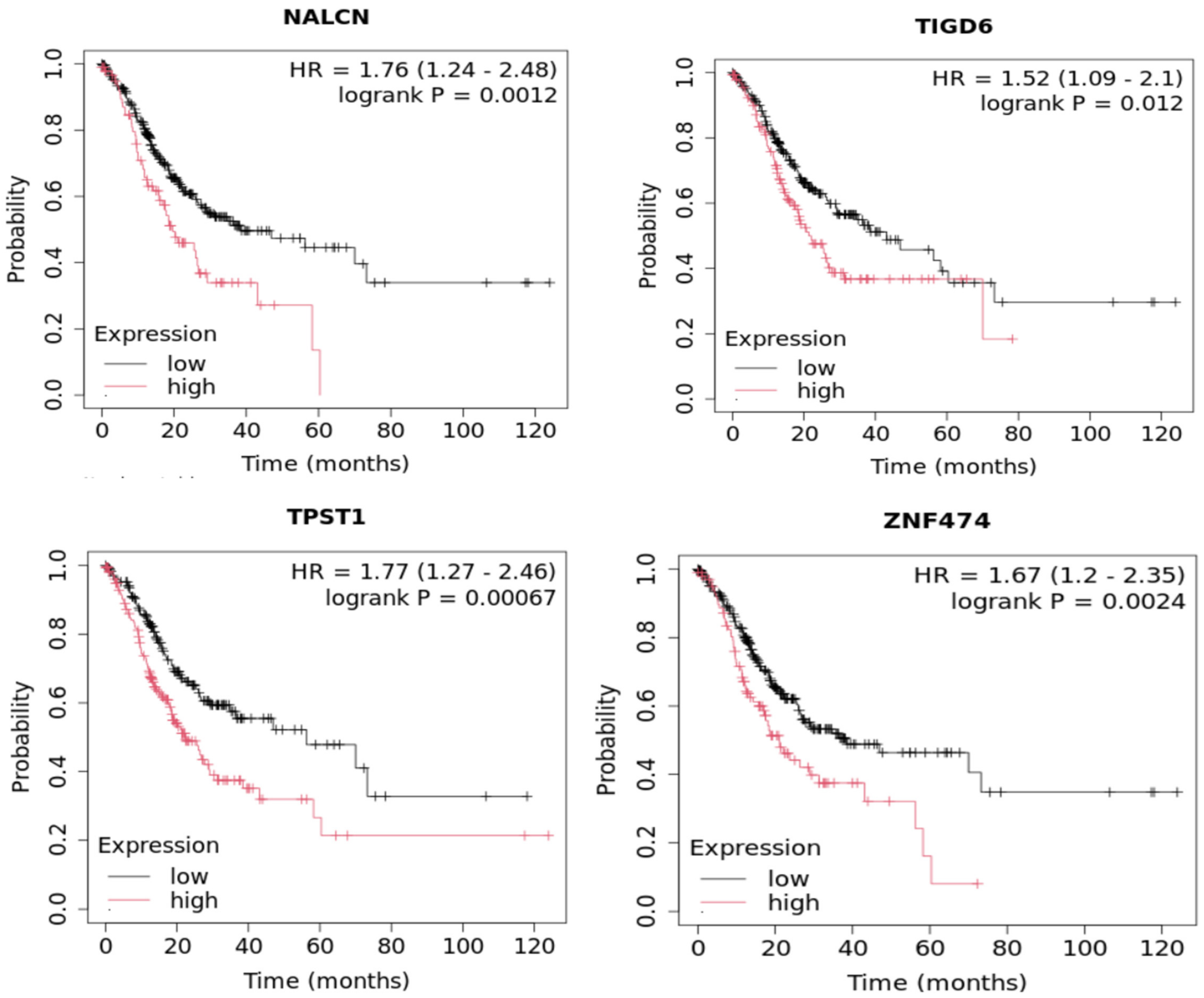

Figure S2. Expression of the 10 novel genes in stomach adenocarcinoma and their prognostic value using the Kaplan Meier Plotter database.

High expression of the 10 novel genes is significantly associated with poor survival in stomach adenocarcinoma.

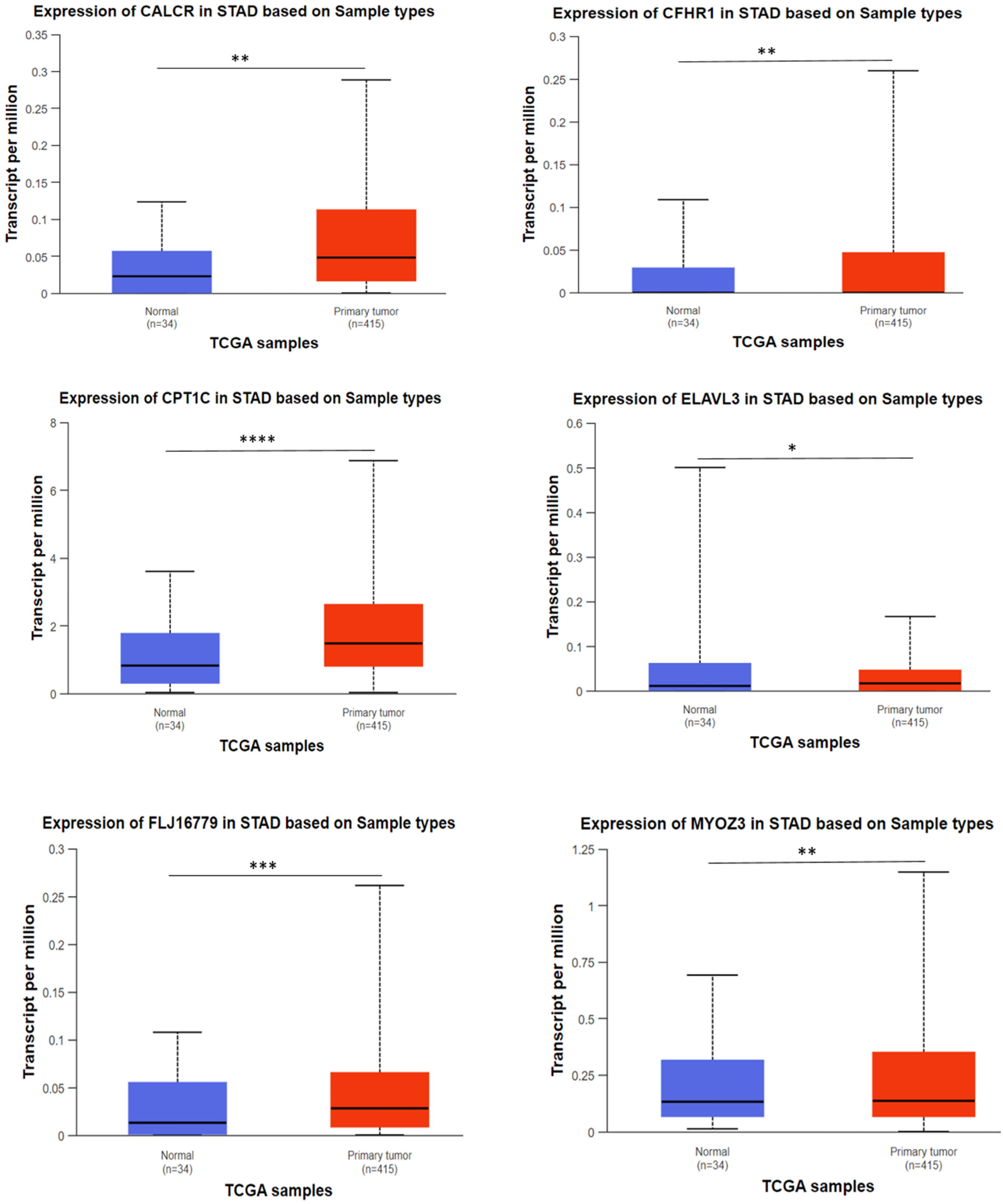
Expression of CALCR in STAD based on Sample types
Transcript per million
0.35
0.3
0.25
0.2
0.15
0.1
0.05
0
**
Normal
(n=34)
Primary tumor
(n=415)
TCGA samples
Expression of CFHR1 in STAD based on Sample types
Transcript per million
0.3
0.25
0.2
0.15
0.1
0.05
0
**
Normal
(n=34)
Primary tumor
(n=415)
TCGA samples
Expression of CPT1C in STAD based on Sample types
Transcript per million
8
6
4
2
0
****
Normal
(n=34)
Primary tumor
(n=415)
TCGA samples
Expression of ELAVL3 in STAD based on Sample types
Transcript per million
0.6
0.5
0.4
0.3
0.2
0.1
0
*
Normal
(n=34)
Primary tumor
(n=415)
TCGA samples
Expression of FLJ16779 in STAD based on Sample types
Transcript per million
0.3
0.25
0.2
0.15
0.1
0.05
0
***
Normal
(n=34)
Primary tumor
(n=415)
TCGA samples
Expression of MYOZ3 in STAD based on Sample types
Transcript per million
1.25
1
0.75
0.5
0.25
0
**
Normal
(n=34)
Primary tumor
(n=415)
TCGA samples

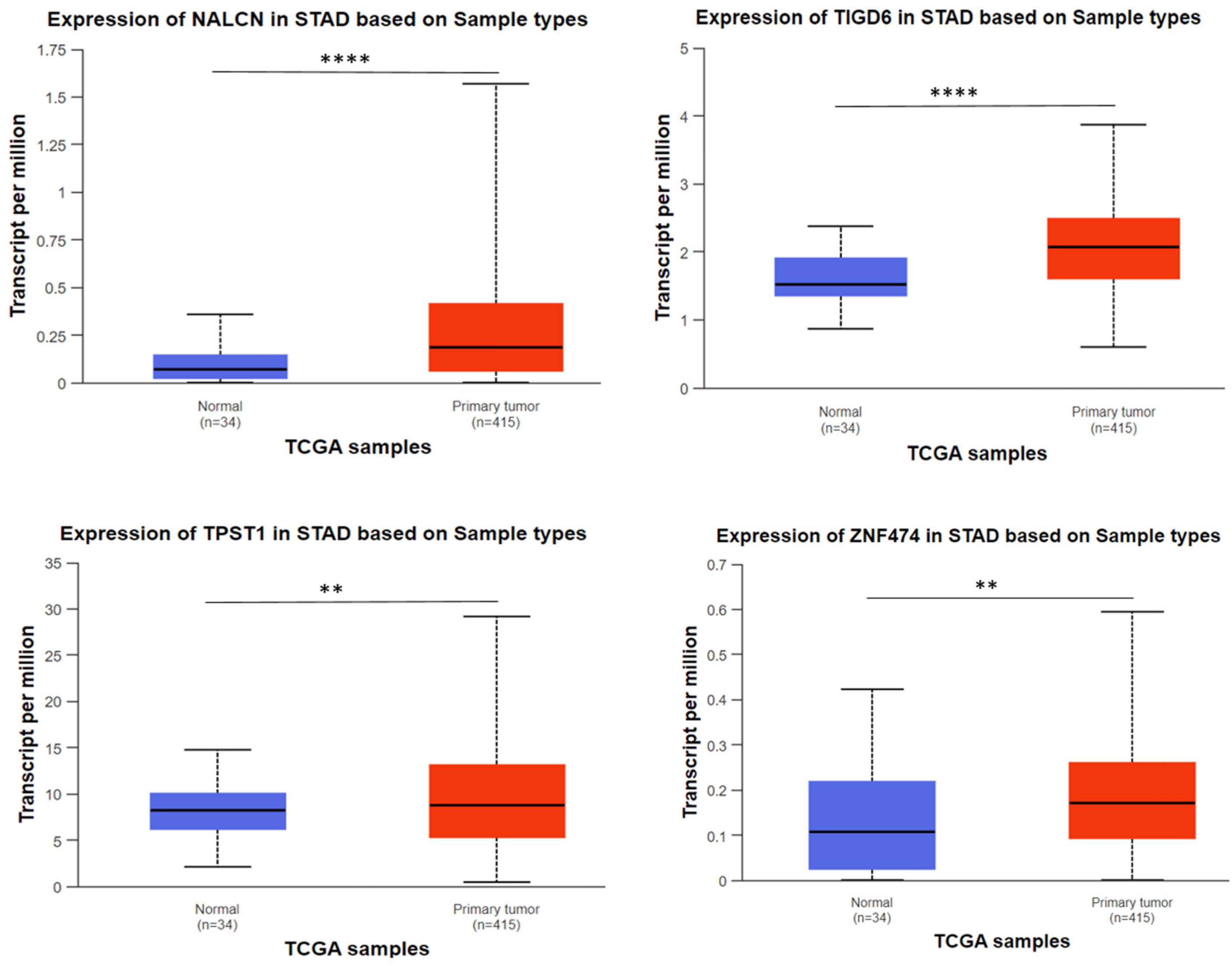

Figure S3. The mRNA expression levels of the 10 novel Prognosis-Associated Genes.

Shows a significant increase in tumor tissues compared to normal controls, determined by UALCAN database.

$*p < 0.05$, $**p < 0.01$, and $***p < 0.001$.

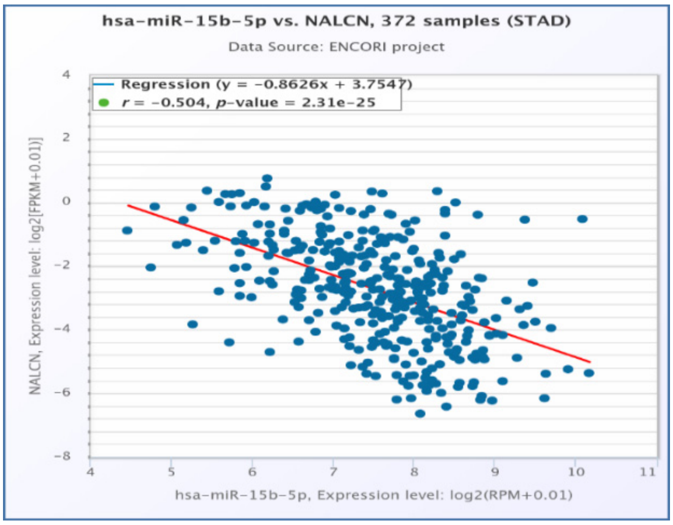
hsa-miR-15b-5p vs. NALCN, 372 samples (STAD)
Data Source: ENCORI project
Regression (y = −0.8626x + 3.7547)
r = −0.504, p-value = 2.31e-25
NALCN, Expression level: log2[FPKM+0.01)]
hsa-miR-15b-5p, Expression level: log2(RPM+0.01)

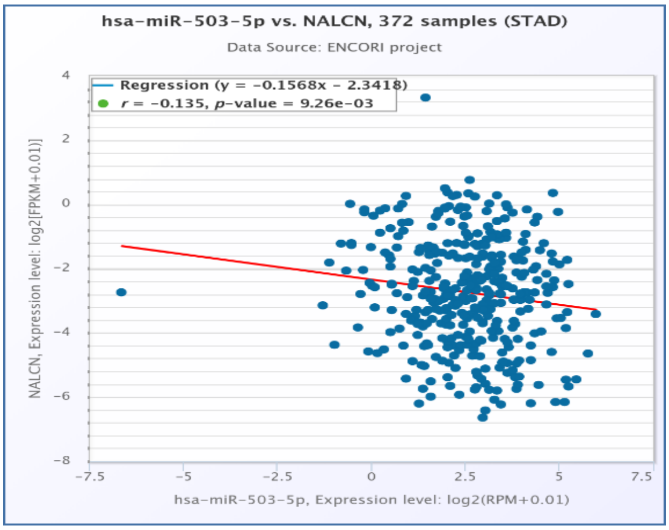
hsa-miR-503-5p vs. NALCN, 372 samples (STAD)
Data Source: ENCORI project
Regression (y = −0.1568x − 2.3418)
r = −0.135, p-value = 9.26e-03
NALCN, Expression level: log2[FPKM+0.01)]
hsa-miR-503-5p, Expression level: log2(RPM+0.01)

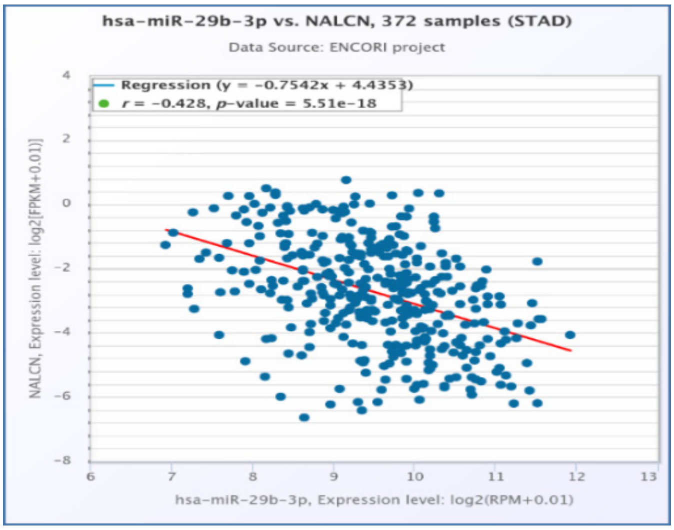
hsa-miR-29b-3p vs. NALCN, 372 samples (STAD)
Data Source: ENCORI project
Regression (y = −0.7542x + 4.4353)
r = −0.428, p-value = 5.51e-18
NALCN, Expression level: log2[FPKM+0.01)]
hsa-miR-29b-3p, Expression level: log2(RPM+0.01)

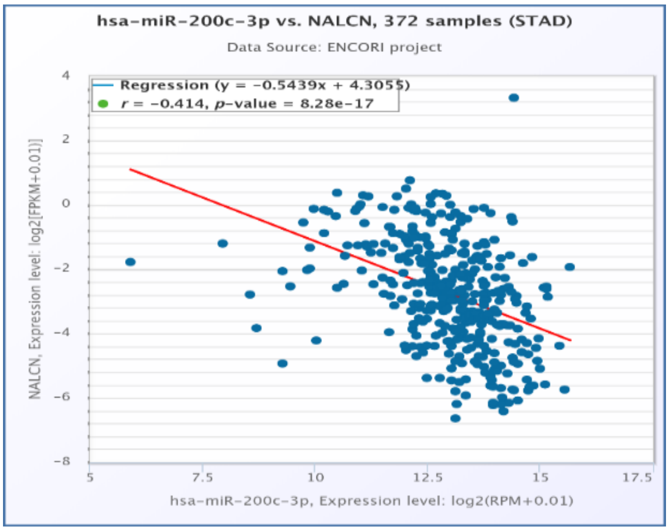
hsa-miR-200c-3p vs. NALCN, 372 samples (STAD)
Data Source: ENCORI project
Regression (y = −0.5439x + 4.3055)
r = −0.414, p-value = 8.28e-17
NALCN, Expression level: log2[FPKM+0.01)]
hsa-miR-200c-3p, Expression level: log2(RPM+0.01)

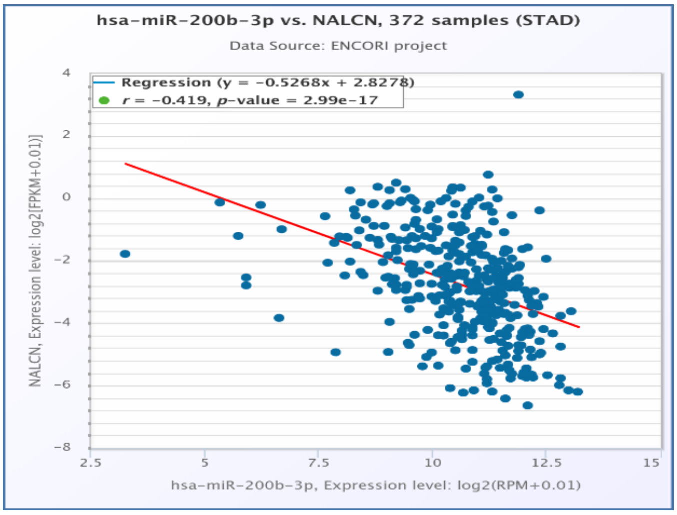
hsa-miR-200b-3p vs. NALCN, 372 samples (STAD)
Data Source: ENCORI project
Regression (y = −0.5268x + 2.8278)
r = −0.419, p-value = 2.99e-17
NALCN, Expression level: log2[FPKM+0.01)]
hsa-miR-200b-3p, Expression level: log2(RPM+0.01)

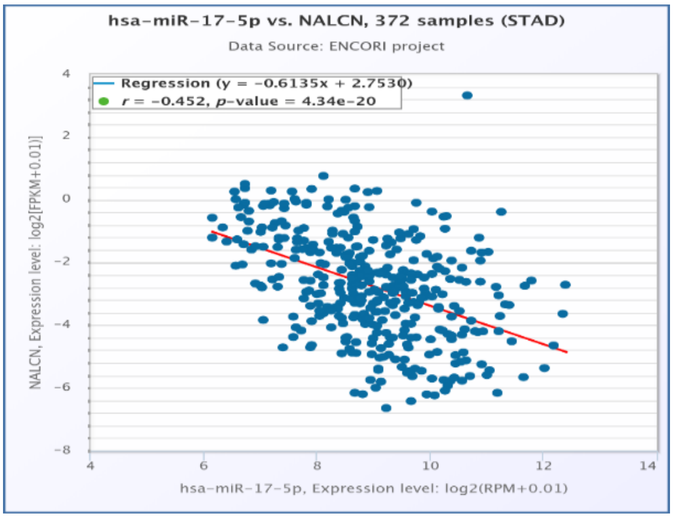
hsa-miR-17-5p vs. NALCN, 372 samples (STAD)
Data Source: ENCORI project
Regression (y = −0.6135x + 2.7530)
r = −0.452, p-value = 4.34e-20
NALCN, Expression level: log2[FPKM+0.01)]
hsa-miR-17-5p, Expression level: log2(RPM+0.01)

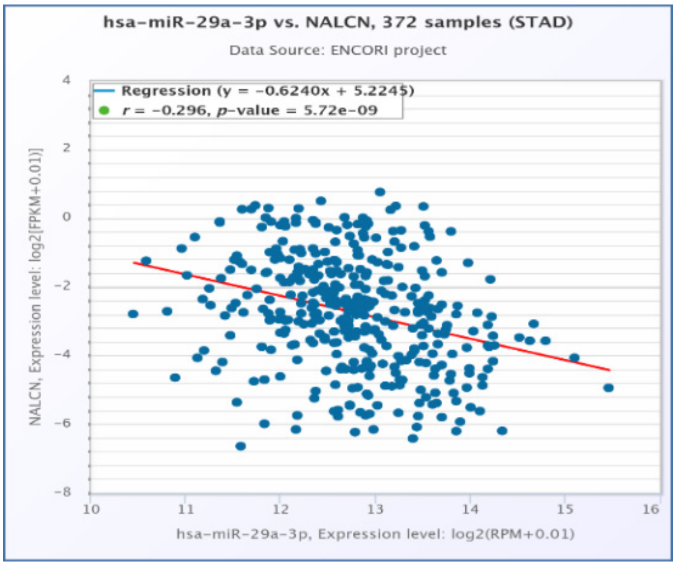
hsa-miR-29a-3p vs. NALCN, 372 samples (STAD)
Data Source: ENCORI project
Regression (y = −0.6240x + 5.2245)
r = −0.296, p-value = 5.72e-09
NALCN, Expression level: log2[FPKM+0.01)]
hsa-miR-29a-3p, Expression level: log2(RPM+0.01)

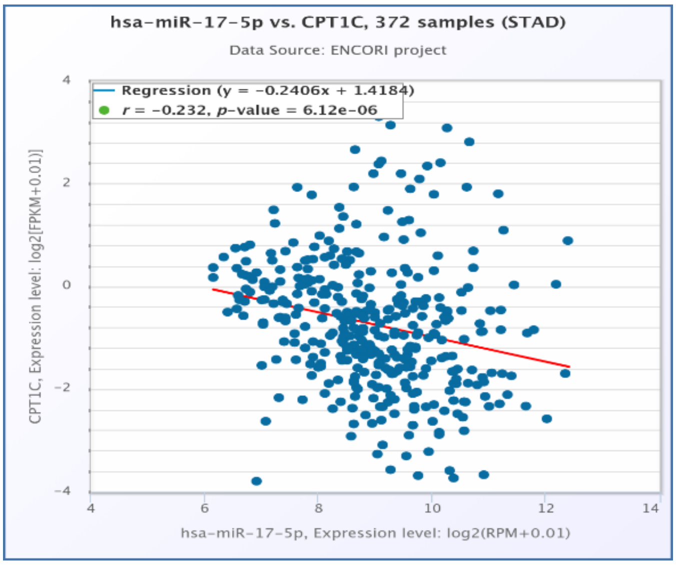
hsa-miR-17-5p vs. CPT1C, 372 samples (STAD)
Data Source: ENCORI project
Regression (y = −0.2406x + 1.4184)
r = −0.232, p-value = 6.12e-06
CPT1C, Expression level: log2[FPKM+0.01)]
hsa-miR-17-5p, Expression level: log2(RPM+0.01)

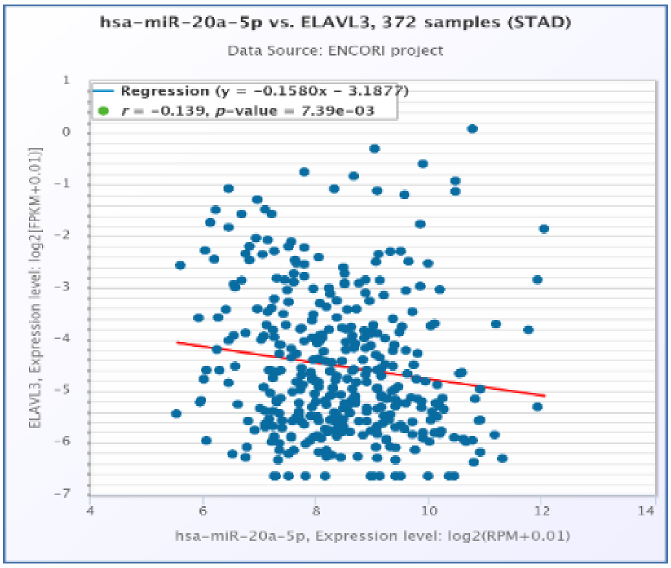
hsa-miR-20a-5p vs. ELAVL3, 372 samples (STAD)
Data Source: ENCORI project
Regression (y = −0.1580x − 3.1877)
r = −0.139, p-value = 7.39e-03
ELAVL3, Expression level: log2[FPKM+0.01)]
hsa-miR-20a-5p, Expression level: log2(RPM+0.01)

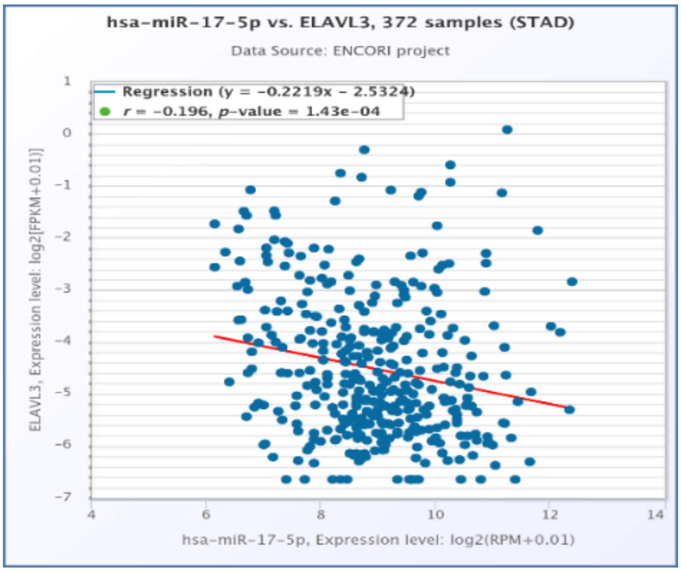
hsa-miR-17-5p vs. ELAVL3, 372 samples (STAD)
Data Source: ENCORI project
Regression (y = −0.2219x − 2.5324)
r = −0.196, p-value = 1.43e-04
ELAVL3, Expression level: log2[FPKM+0.01)]
hsa-miR-17-5p, Expression level: log2(RPM+0.01)

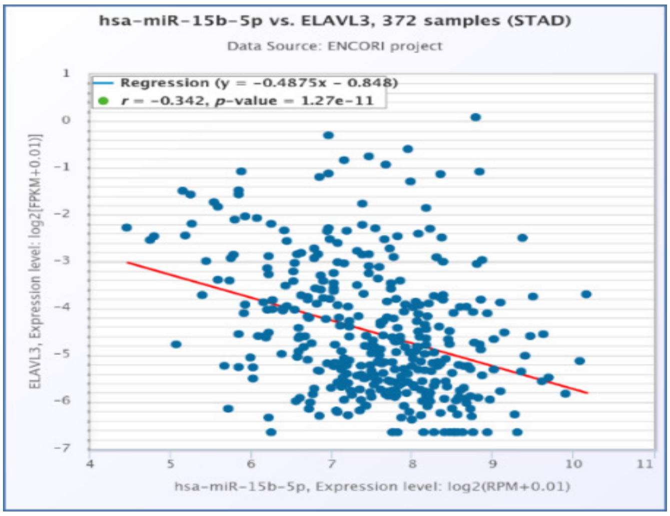
hsa-miR-15b-5p vs. ELAVL3, 372 samples (STAD)
Data Source: ENCORI project
Regression (y = −0.4875x − 0.848)
r = −0.342, p-value = 1.27e-11
ELAVL3, Expression level: log2[FPKM+0.01)]
hsa-miR-15b-5p, Expression level: log2(RPM+0.01)

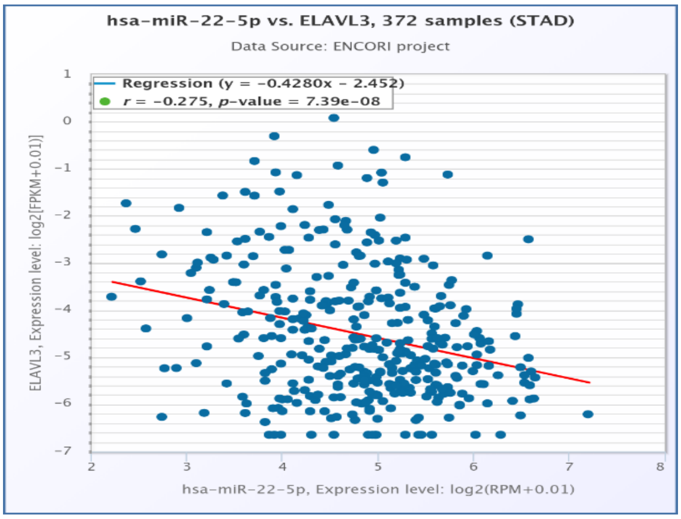
hsa-miR-22-5p vs. ELAVL3, 372 samples (STAD)
Data Source: ENCORI project
Regression (y = −0.4280x − 2.452)
r = −0.275, p-value = 7.39e-08
ELAVL3, Expression level: log2[FPKM+0.01)]
hsa-miR-22-5p, Expression level: log2(RPM+0.01)

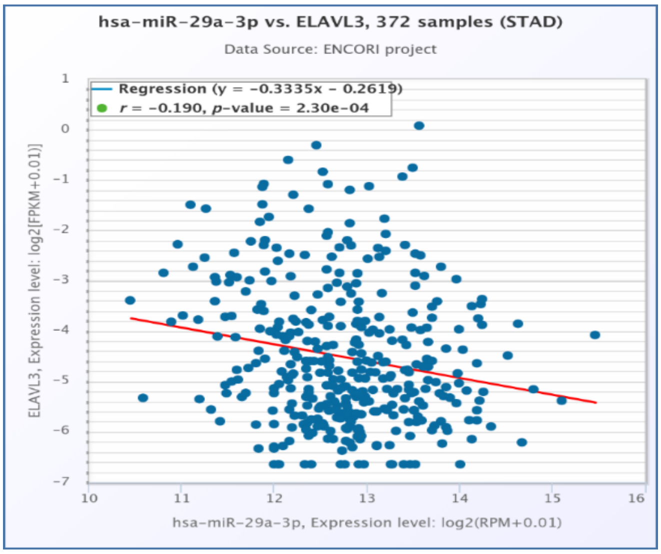
hsa-miR-29a-3p vs. ELAVL3, 372 samples (STAD)
Data Source: ENCORI project
Regression (y = -0.3335x - 0.2619)
r = -0.190, p-value = 2.30e-04
ELAVL3, Expression level: log2[FPKM+0.01)]
hsa-miR-29a-3p, Expression level: log2(RPM+0.01)

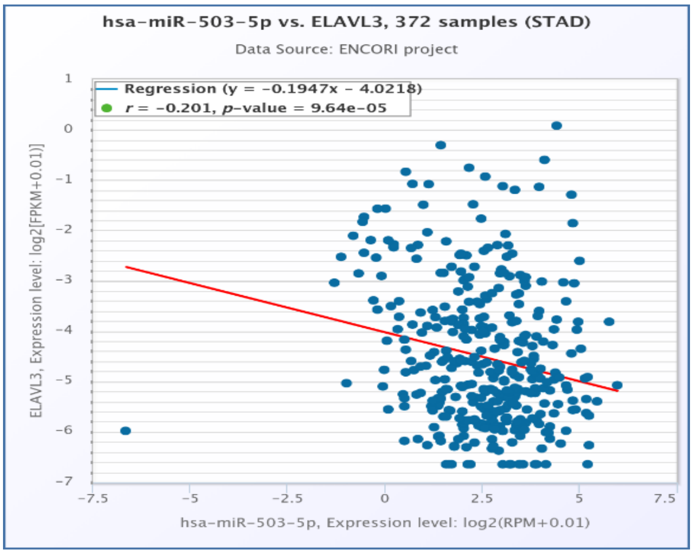
hsa-miR-503-5p vs. ELAVL3, 372 samples (STAD)
Data Source: ENCORI project
Regression (y = -0.1947x - 4.0218)
r = -0.201, p-value = 9.64e-05
ELAVL3, Expression level: log2[FPKM+0.01)]
hsa-miR-503-5p, Expression level: log2(RPM+0.01)

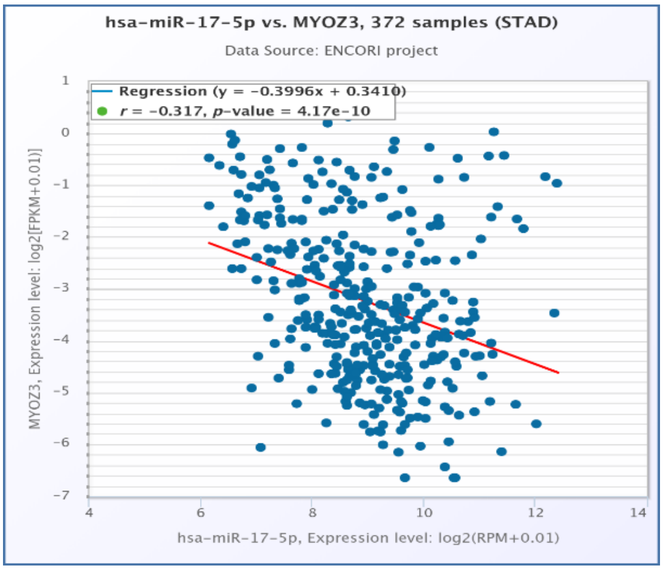
hsa-miR-17-5p vs. MYOZ3, 372 samples (STAD)
Data Source: ENCORI project
Regression (y = -0.3996x + 0.3410)
r = -0.317, p-value = 4.17e-10
MYOZ3, Expression level: log2[FPKM+0.01)]
hsa-miR-17-5p, Expression level: log2(RPM+0.01)

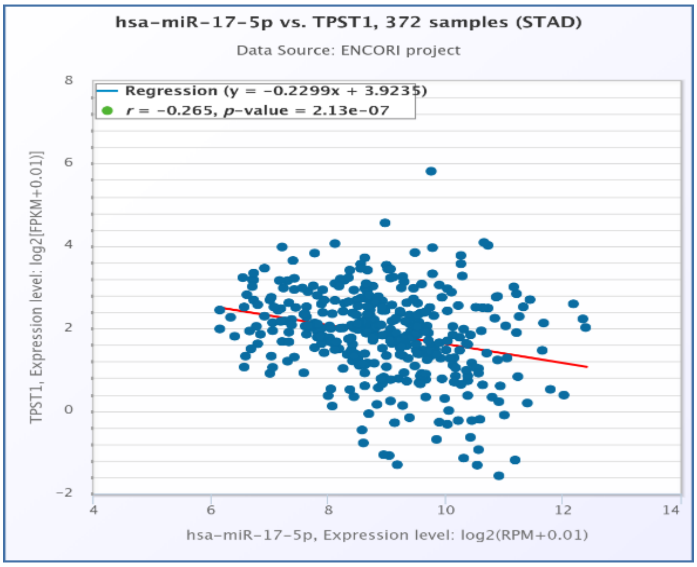
hsa-miR-17-5p vs. TPST1, 372 samples (STAD)
Data Source: ENCORI project
Regression (y = -0.2299x + 3.9235)
r = -0.265, p-value = 2.13e-07
TPST1, Expression level: log2[FPKM+0.01)]
hsa-miR-17-5p, Expression level: log2(RPM+0.01)

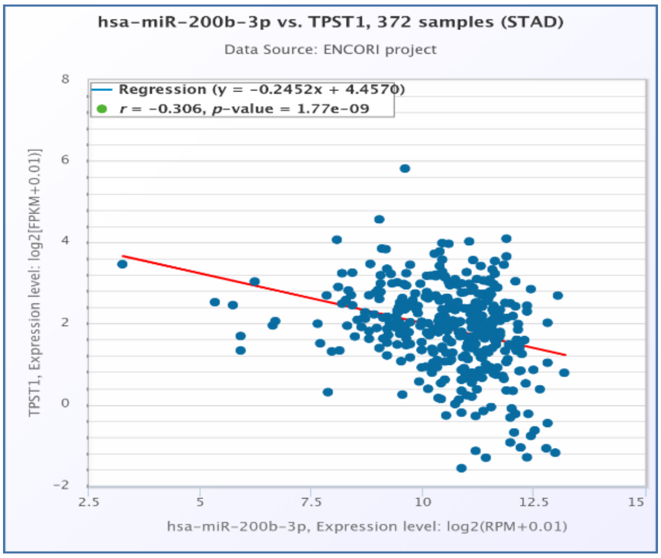
hsa-miR-200b-3p vs. TPST1, 372 samples (STAD)
Data Source: ENCORI project
Regression (y = -0.2452x + 4.4570)
r = -0.306, p-value = 1.77e-09
TPST1, Expression level: log2[FPKM+0.01)]
hsa-miR-200b-3p, Expression level: log2(RPM+0.01)

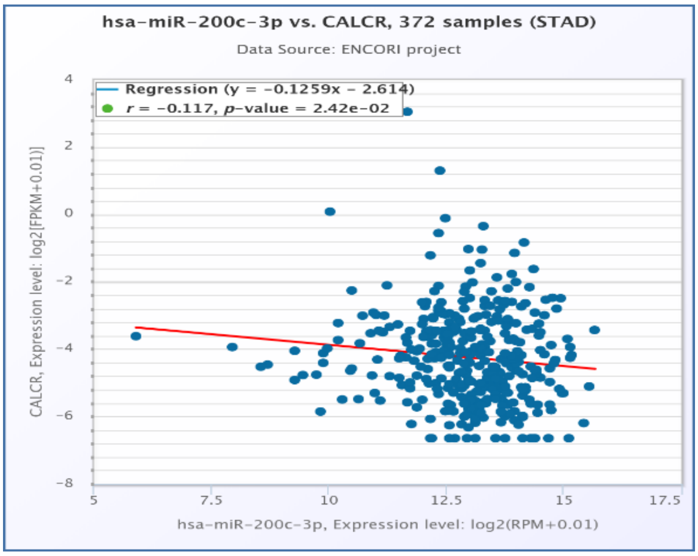
hsa-miR-200c-3p vs. CALCR, 372 samples (STAD)
Data Source: ENCORI project
Regression (y = -0.1259x - 2.614)
r = -0.117, p-value = 2.42e-02
CALCR, Expression level: log2[FPKM+0.01)]
hsa-miR-200c-3p, Expression level: log2(RPM+0.01)

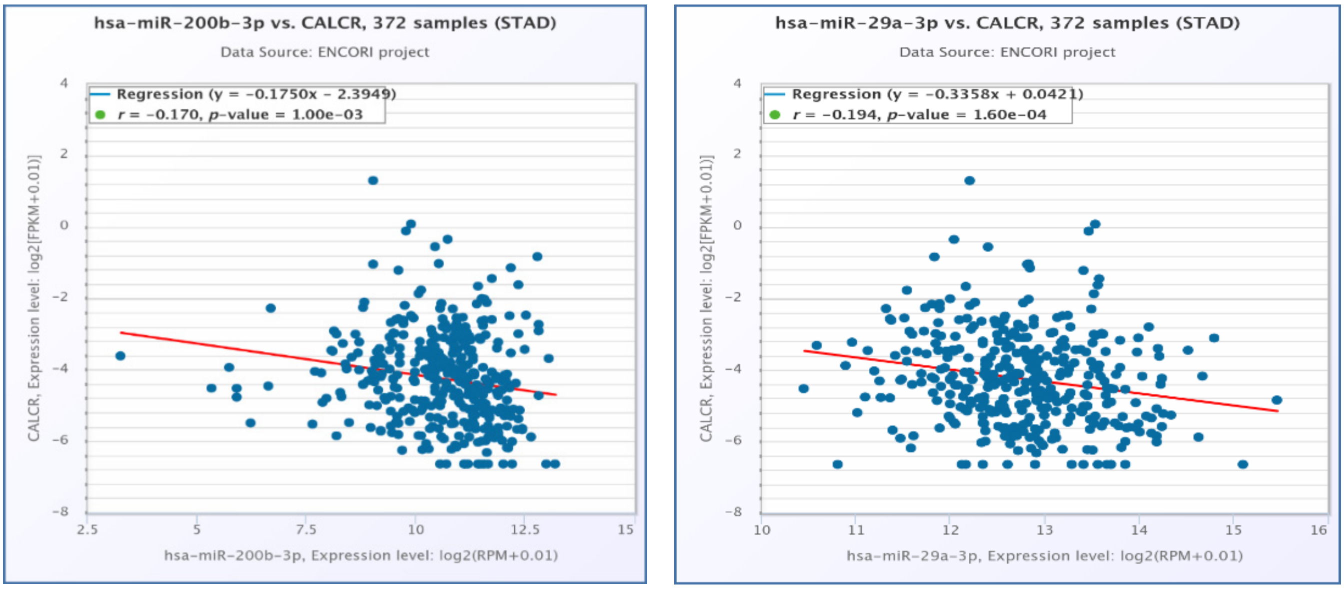

Figure S4. Expression correlation between mRNA and miRNA.

Expression of NALCN mRNA was significantly negatively associated with hsa-mir-15b-5p(A), hsa-mir-503-5p(B), hsa-mir-29b-3p(C), hsa-mir-200c-3p(D), hsa-mir-17-5p(E), hsa-mir-29a-3p(F), hsa-mir-200b-3p (G) and hsa-mir-20a-5p(H) expression. Expression of CPT1C mRNA was significantly negatively associated with hsa-mir-17-5P(I). Expression of ELAVL3 mRNA was significant negative expression with hsa-mir-20a-5p(J), hsa-mir-17-5p(K), hsa-mir-15b-5p(L), hsa-mir-22-3p(M), hsa-mir-29a-3p(N), and hsa-mir-503-5p(O). Expression of MYOZ3 mRNA was significantly negatively associated with hsa-mir-17-5p(P). hsa-mir-17-5p(Q) and hsa-mir-29c-3p(R) were negatively associated with TPST1mRNA expression. The CALCR mRNA was significantly negatively associated with hsa-mir-200c-3p(S), hsa-mir-200b-3p (T), and hsa-mir-29a-3p (U).
